# Hole-Transporting Materials for Printable Perovskite Solar Cells

**DOI:** 10.3390/ma10091087

**Published:** 2017-09-15

**Authors:** Paola Vivo, Jagadish K. Salunke, Arri Priimagi

**Affiliations:** Laboratory of Chemistry and Bioengineering, Tampere University of Technology, P.O. Box 541, FI-33101 Tampere, Finland; jagdishsalunke.nmu@gmail.com (J.K.S.); arri.priimagi@tut.fi (A.P.)

**Keywords:** perovskite solar cells, hole-transporting material, printable, small-molecule, polymer, inorganic, hybrid

## Abstract

Perovskite solar cells (PSCs) represent undoubtedly the most significant breakthrough in photovoltaic technology since the 1970s, with an increase in their power conversion efficiency from less than 5% to over 22% in just a few years. Hole-transporting materials (HTMs) are an essential building block of PSC architectures. Currently, 2,2’,7,7’-tetrakis-(*N*,*N’*-di-*p*-methoxyphenylamine)-9,9’-spirobifluorene), better known as spiro-OMeTAD, is the most widely-used HTM to obtain high-efficiency devices. However, it is a tremendously expensive material with mediocre hole carrier mobility. To ensure wide-scale application of PSC-based technologies, alternative HTMs are being proposed. Solution-processable HTMs are crucial to develop inexpensive, high-throughput and printable large-area PSCs. In this review, we present the most recent advances in the design and development of different types of HTMs, with a particular focus on mesoscopic PSCs. Finally, we outline possible future research directions for further optimization of the HTMs to achieve low-cost, stable and large-area PSCs.

## 1. Introduction

The world demand for energy is growing rapidly and continuously. In 2016, the total worldwide energy consumption was approximately 1.33 × 10^8^ tonnes of oil equivalent (toe) [1], which corresponds to roughly 1.5 × 10^5^ terawatt hours (TWh). These numbers continuously rise, due to the growth of the population and the world economy. Hence, there is no other clever option to meet the future energy demands than to invest in environmentally-clean energy resources, such as solar energy. Every year the Sun provides the Earth’s surface with 1.9 × 10^8^ TWh of radiation [2]; thus, it supplies in about 7 h enough energy to fulfill the world energy needs for one year. Sunlight is free and unlimited. Moreover, solar energy is a clean, renewable, readily available and ubiquitous energy source. The large gap between the current use of solar energy and its unexploited potential represents a great challenge in energy research.

Conventional crystalline silicon-based solar cells are present in still 90% of commercial photovoltaic devices. However, there is a need to replace silicon photovoltaics (PV) with low-cost, easy-to-assemble, flexible and lightweight devices. Recently, hybrid organic-inorganic perovskite solar cells (PSCs) became in just a few years one of the most exciting PV technologies. They have a huge potential to dominate the photovoltaic market, being at the same time highly efficient, low-cost and compatible with inexpensive fabrication processes such as inkjet printing. However, despite the impressive advances in their power conversion efficiencies over the last few years, PSCs still suffer major stability issues (lifetime <1 year), which hinder their commercialization. The PSCs’ lifetime may be enhanced by optimizing different parts of the device and its constituents [3]: perovskite crystal structure, cell encapsulation, film quality, alternative designs, conducting layers and interfaces.

In this review, we focus on the efforts to propose alternative charge (electrons and holes) conducting layers and particularly the hole-transporting materials (HTMs), to enhance the stability and cost effectiveness without impacting the power conversion efficiency (PCE). A few other reviews are already published on the topic of HTMs in PSCs [4,5,6,7]. The perovskite research field is very dynamic, with a continuous feed of new HTMs. Our paper aims to assess the most recent works, with a particular emphasis on the chemistry of the HTMs and the correlation between the molecular design, efficiency, stability and cost-effectiveness of PSCs.

In Section 2, we give an overview of PSCs in terms of photovoltaic architectures and current limitations of this technology. In Section 3, we summarize the cutting-edge and most recent achievements related to HTMs for printable PSCs, focusing on different types of HTMs, i.e., organic, inorganic and hybrid ones. The surveyed works will mostly refer to mesoscopic PSCs. The interested reader can consult several other papers for a comprehensive overview of the HTMs in planar architectures [4,5,8]. Finally, we assess the most promising research directions among the different HTM categories, and we highlight how HTMs could be further optimized to enhance the stability and cost effectiveness of printable PSCs.

## 2. Perovskite Solar Cells

PSCs are the fastest growing technology in solar cell research. They have received tremendous interest from the scientific community, due to a five-fold increase in their PCEs in just three years [9,10,11]. The current PCE (certified) record, set at 22.1% [12], is already close to that achieved after decades of development of traditional silicon photovoltaics (25.3%) [11]. Furthermore, compared to the heavy and rigid silicon solar cells, rolls of perovskite films have the potential advantages of being inexpensive, light, bendable and aesthetically attractive.

**Figure 1:**
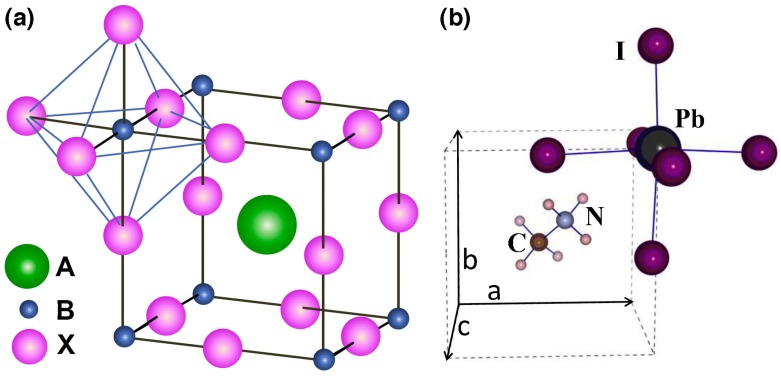
(**a**) ABX_3_ perovskite structure showing the BX_6_ octahedral and larger A cation occupied in the cubo-octahedral site; (**b**) unit cell of cubic CH_3_NH_3_PbI_3_ perovskite (reproduced with permission from [13], published by Elsevier under the terms of the Creative Commons Attribution-Non Commercial-No Derivatives Licence (CC BY NC ND)).

PSCs are based on halide perovskite materials, a class of compounds with the general chemical formula ABX_3_, where X is either oxygen or halogen (anion) and A and B are cations, A being larger than B. The A cation occupies a cubo-octahedral site shared with twelve X anions, while the B cation is stabilized in an octahedral site shared with six X anions (Figure 1) [13].

In the formula, A is typically a small organic cation like CH_3_NH_3_^+^ (methylammonium), C_2_H_5_NH_3_^+^ (ethylammonium) or HC(NH_2_)_2_^+^ (formamidinium). Cation A has a +1 charge, and it is the most important component of the perovskite molecule, determining the structure and the crystal size of perovskite and thus directly influencing the stability and the optoelectronic properties of the perovskite [14]. B is usually a metal ion with a charge of +2 like Pb^2+^, Sn^2+^ or Cu^2+^, and X is usually Cl^−^, Br^−^ or I^−^.

Perovskite materials have exceptional properties resulting in high-performance solar cells. These properties include a remarkably high absorption over the visible and near-infrared spectrum, low exciton binding energy, charge carrier diffusion lengths in the μm range (1 μm in thin films [15] and up to 175 μm in single crystals [16]), a sharp optical band edge and a tunable band gap by replacing the cations and anions in the perovskite structure.

### 2.1. Mesoscopic and Planar Architectures

The first perovskite-based solar cells stemmed from dye-sensitized solar cells (DSSCs) where the perovskite nanocrystals were used as the sensitizer [17]. Their functioning principle resembled that of the dye in DSSCs. Later, the ambipolar behavior of perovskite was understood: a few hundred nanometer-thick layers of perovskite are sufficient to perform efficient charge generation and transport. In other words, perovskite can work as a light absorber, as well as an electron and hole conductor, all at the same time [18]. Such a discovery enabled numerous new configurations and materials for PSCs, among which two architectures emerged, namely mesoscopic and planar structures.

**Figure 2:**
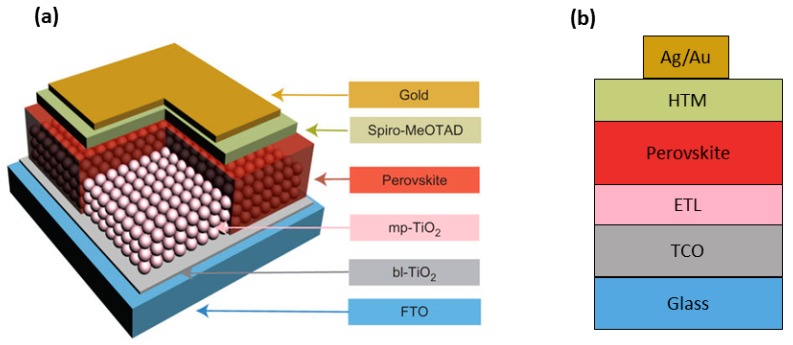
Schematic illustration of the (**a**) mesoscopic and (**b**) planar perovskite solar-cell configurations. In the mesoscopic architecture (a), a smooth perovskite capping layer covers the top of the mesoporous TiO_2_ layer. The hole-transporting material (HTM), typically 2,2’,7,7’-tetrakis-(*N*,*N’*-di-*p*-methoxyphenylamine)-9,9’-spirobifluorene) (spiro-OMeTAD), is spin-coated atop the perovskite film. The most frequent film thicknesses reported for the layers of perovskite solar cell (PSC) structures are 50 nm (blocking TiO_2_ layer, bl-TiO_2_), 300 nm (mesoporous TiO_2_, mp-TiO_2_), 4–500 nm (perovskite), 1–200 nm (spiro-OMeTAD capping layer) and 80 nm (gold/silver). Please note that a systematic layer thickness optimization is still missing in the literature. In the planar configuration (b), the perovskite film is deposited directly on top of the electron-transporting layer (ETL), commonly a TiO_2_ dense hole-blocking layer (the original figure in (a) was reproduced with permission from [24], published by Nature Publishing Group). FTO, fluorine-doped tin oxide; TCO, transparent conducting oxide.

The mesoscopic PSC is the most widely-adopted geometry in research labs, because of the ease of fabrication and outstanding record efficiencies [19]. However, very recently, more and more studies have been reported where a planar heterojunction PSC is proposed [20], thus making it possible to fabricate highly-efficient PSCs also with planar architectures (Figure 2) [21]. The traditional mesoscopic perovskite cell architecture utilizes a thin (30–50 nm) and compact hole blocking layer between the transparent conducting oxide (TCO) layer (mostly fluorine-doped tin oxide (FTO) coating) and a mesoporous scaffold [22,23]. The perovskite is infiltrated into the mesoporous metal oxide scaffold, typically made of either n-type material such as TiO_2_ and ZnO or an insulating dielectric oxide like Al_2_O_3_. The role of the scaffold is two-fold: to facilitate the formation of a homogeneous film on a large area forming a junction of low ohmic resistance and to enhance the electron transfer [18].

The light-harvesting perovskite layer is typically spin-coated from *N,N-*dimethyl-formamide (DMF) or dimethyl sulfoxide (DMSO). A hole-transporting material (HTM) layer is deposited atop, and the structure is completed by a metal contact (typically Au or Ag) [25].

**Figure 3:**
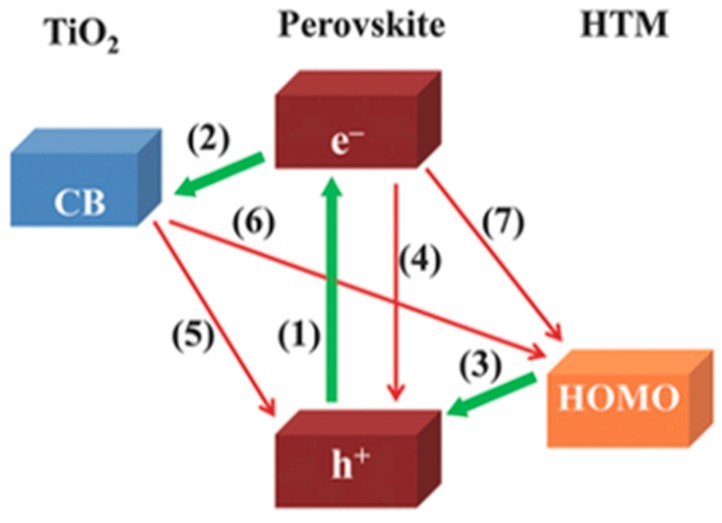
Charge-transfer processes in perovskite solar cells. The valence band energy and the conduction band (CB) of methyl ammonium lead iodide (MAPbI_3_) perovskite are at −5.43 eV and −3.7 eV, respectively. The CB of TiO_2_ lies at −4.2 eV, and the HOMO energy level of the widely-used HTM spiro-OMeTAD is at −5.22 eV [5] (reproduced with permission from [5], published by John Wiley & Sons, Inc.).

An overview of the key charge-transfer processes taking place in PSCs is depicted in Figure 3 [5]. The photoexcitation (green arrow marked with (1) in Figure 3) generates electrons and holes in the perovskite absorber layer: the first are injected into titania (green arrow marked with (2) in Figure 3), and the second are transferred to the HTM (green arrow marked with (3) in Figure 3). Finally, charges are collected at the respective electrodes. Undesired recombination (red arrows marked with (4), (5) and (7) in Figure 3) can take place at the TiO_2_|perovskite|HTM interfaces [5]. The determination of the time scale and charge carrier dynamics taking place at different interfaces in PSC is of crucial importance for being able to further optimize the devices [18]. Surprisingly, only a few research groups have so far investigated different types of time-resolved techniques to reveal the PSC photophysics [26,27,28]. Hence, more research on this topic is needed.

The planar PSC architecture, though attractive due to the simple and straightforward preparation, presents still several drawbacks when compared to its mesoscopic counterpart in terms of performance and hysteresis [29]. Planar PSCs usually employ a p-i-n configuration where poly(3,4-ethyl-enedioxythiophene):poly(styrenesulfonate) (PEDOT:PSS) and 6,6-phenyl C61-butyric acid methyl ester (PCBM) are mostly adopted as hole-transport and electron-transport layers (ETL), respectively (Figure 2). Alternatively, an n-i-p configuration is used in planar PSCs, where TiO_2_ is the typical ETL [30].

The microstructure of the perovskite layer within mesoscopic and planar architectures is significantly different. In the mesoscopic assembly, the perovskite grain growth is restricted by the pore size of the mesoporous scaffold, whereas in the planar architecture, the perovskite grain growth is solely limited by the growth of neighboring perovskite grains. Recently, the correlation between microstructure and charge transport/recombination kinetics of the two architectures has been established [31].

### 2.2. Current Challenges of Perovskite Solar Cells Research

Since the first pioneering works on perovskite-sensitized solar cells in 2009–2012, there has been a huge surge of interest in perovskite solar cells [32], leading to the so-called Perovskite Fever [33]. However, there are certainly serious challenges to be addressed when thinking to bring PSCs from the laboratory to the market, namely the enhancement of the device stability, the replacement of lead (Pb) in perovskite materials to overcome the toxicity issues and the upscaling of lab-sized cells to larger modules [34,35]. In fact, a technology impediment to PSC commercialization lies in the current methods for fabricating high-efficiency PSCs, such as spin-coating or thermal evaporation, which are not compatible with large-scale production processes, such as the roll-to-roll (R2R) process.

Another issue related to the poor reproducibility of PSCs is the presence of hysteresis in the voltage-dependent photocurrent, which complicates the determination of the real solar-to-electrical power conversion efficiency of the devices [36].

Among all of the above-mentioned challenges on the road to the commercialization of PSCs, stability is however the main impediment. While silicon cells last for 25 years, typical high-efficiency PSCs, when unencapsulated, can last for a few months. The stability issues related to PSCs [37] are: (1) the degradation of the perovskite absorber layer due to moisture penetration; (2) the poor interface between hybrid layers; and (3) the instability of individual deposited layers, mostly the HTMs. Thus, one of the hottest challenges in PSCs research is the discovery of HTMs that are both efficient and cost effective at the same time, so as to enhance the solar cells’ lifetime while reducing their fabrication costs. One key-approach to minimize the effects of moisture, oxygen and thermal influence on the HTMs’ degradation is to design materials with hydrophobic functionalities, e.g., fluoroaromatics or fluoroaliphatics. These are responsible not only for the increase in the air/oxygen stability of PSCs but also for the enhancement of the thermal stability of the materials themselves. Fluorination is a great strategy to introduce air stability and chemical stability in organic compounds, by lowering both the HOMO and LUMO in small molecules, as well as in polymers. Furthermore, fluorination profoundly affects the materials’ packing in solid state, thereby tuning their charge-transport properties.

## 3. Hole-Transporting Materials

The HTM plays a key-role in the PSC structures, being deposited in the heart of the cell between the perovskite layer and the evaporated metal electrode. Its two-fold role is to:

- prevent the direct contact between the perovskite and the metal contact, which minimizes charge recombination and avoids degradation at the metal-perovskite interface;
- extract positive charges (holes) from perovskite and transport them to the top-electrode.

Ideally, HTMs must fulfil several general requirements to enhance the efficiency of the PSC [4]. First, the highest occupied molecular orbital (HOMO) energy level of the HTM should lie above the valence band energy of perovskite. A shift of the HOMO level towards the perovskite will result in an enhancement in the open circuit voltage of the PSCs (Figure 3) [4]. To be more exact, what drives the hole capture is the difference between the Fermi level of the hole transporter and that of the holes in the perovskite under illumination [4]. Moreover, a good hole-mobility (ideally >10^−3^ cm^2^ V^−1^ s^−1^), as well as thermal and photochemical stability are required characteristics of an HTM [5]. Furthermore, transparency in the visible spectrum is desirable to avoid the absorption screen effect toward the active materials/absorber. In order to avoid crystallization, an amorphous phase with a glass transition temperature above 100 °C is also required. In the case of the mesoscopic architecture, adequate HTM pore filling is needed, as well. This can be more easily obtained with small-molecule HTMs. Finally, the thickness optimization of the HTM layer is also important to minimize the increase in the series resistance, which directly correlates with the cell fill factor (FF) reduction. Usually, an optimal thickness of the HTM lies within the 100–200 nm range.

**Figure 4:**
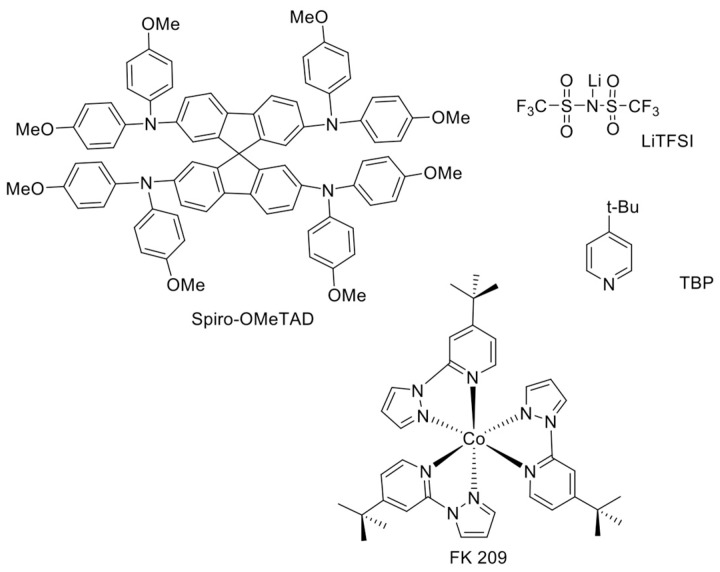
Molecular structures of spiro-OMeTAD, the most widely-used HTM in PSCs, and its dopants (lithium bis(trifluoromethanesulfonyl) imide salt (LiTFSI), 4-tert-butylpyridine (TBP), tris(2-(1*H*-pyrazol-1-yl)-4-*tert*-butylpyridine)cobalt(III) tri[bis(trifluoromethane)sulfonimide] (FK 209).

The most widely-explored compounds for HTM applications are certainly those based on the triphenylamine (TPA) moiety. In particular, 2,2’,7,7’-tetrakis-(*N*,*N’*-di-*p*-methoxyphenylamine)-9,9’-spirobifluorene), or spiro-OMeTAD, is a TPA-based HTM adopted in the great majority of the works, first on solid-state DSSCs [38] and later on PSCs, as well (Figure 4). The reason why spiro-OMeTAD is still the most widely-used HTM is that it leads to very efficient PSCs. The PCEs achieved with spiro-OMeTAD have rarely been achieved or exceeded with other HTMs.

Nevertheless, there are serious drawbacks of spiro-OMeTAD that preclude it from being the definitive HTM in PSCs, such as:

- high costs: spiro-OMeTAD is prohibitively expensive (~500 $/g) [39] because of (a) its onerous multistep synthesis that requires a low temperature (−78 °C); (b) the sensitive (n-butyllithium or Grignard reagents) and aggressive (Br_2_) reagents involved in the synthetic scheme [40]; (c) the costly sublimation step required for purification [41].
- negative impact on stability: the use of spiro-OMeTAD limits the long-term stability of the devices [39], thus inhibiting the upscaling application in the photovoltaic industry.
- sub-optimal charge transport: when in pristine form, spiro-OMeTAD shows a modest hole-mobility and conductivity (1.67 × 10^−5^ cm^2^ V^−1^ s^−1^ and 3.54 × 10^−7^ S cm^−1^, respectively) [42], thus requiring additives and chemical p-dopants to enhance the hole conductivity by 10-fold and thus the conversion efficiency of PSCs.

The dopants typically incorporated into spiro-OMeTAD solution are lithium bis(trifluoromethanesulfonyl) imide salt (LiTFSI) [43], 4-tert-butylpyridine (TBP) [43], and cobalt (III) complexes such as tris(2-(1*H*-pyrazol-1-yl)-4-*tert*-butylpyridine)cobalt(III) tri[bis(trifluoromethane)sulfonimide] (Co[t-BuPyPz]3[TFSI]3), coded as FK 209 [44]. Their molecular structures are shown in Figure 4. While on the one hand, the dopants are crucial for the enhancement of spiro-OMeTAD performance in terms of higher conductivity, better electron injection and retarded recombination, on the other hand, their use can negatively impact the stability of the devices [45].

In the last few years, a wide range of HTMs has been proposed as alternatives to spiro-OMeTAD in PSCs, based both on organic and inorganic compounds [5,8]. However, only very few examples allowed reaching or surpassing the power conversion efficiency (PCE) of 20% [10,12,39,46,47], which is comparable with the efficiencies obtained with spiro-OMeTAD [48,49,50]. Currently, the champion PSC with world record efficiency includes the polymeric HTM poly-[bis(4-phenyl)(2,4,6-trimethylphenyl)amine] (PTAA) [12].

It is also important to mention the work by Mei et al., which contains one of the rare examples of an HTM-free perovskite solar cell [51]. In fact, perovskite can function as the light absorber and hole transporter, as well. Despite the modest efficiency achieved by the HTM-free configuration (12.8%), the work reported in [51] presents a highly stable solar cell structure able to last over 1000 h in ambient conditions under full sunlight illumination. The heart of this fully-printable architecture is a double layer of mesoporous ZrO_2_ and TiO_2_ scaffold infiltrated with perovskite, which allows low defect concentration in the perovskite crystallization, as well as better contact with the TiO_2_ scaffold.

In this section, the most interesting examples for each category of HTMs, namely organic, inorganic and hybrid, are reviewed. However, due to the extremely intense research on the topic, it will not be possible to present all of the numerous works on HTMs, but only a relatively small selection of them. From this section, the reader can have a picture of different classes of HTMs, among those which are solution-processable and thus suitable for printable solar cells.

It is worth noting that a straightforward comparison between the efficiencies of the many HTMs presented is not possible, as the cell performances are closely related to many factors (e.g., operating conditions, optimization of the other constituents of the PSC, measurements’ accuracy, etc.), which vary from lab to lab. That is why in all of the examples, it has been always important to compare the figure of merits of the devices based on the target HTM with those of the control cells containing spiro-OMeTAD. Furthermore, very recently, mixed-ion formamidinium lead iodide—methyl ammonium lead bromide (FAPbI_3_)_1−x_(MAPbBr_3_) perovskites have been introduced, with a significant boost in the device PCEs. Hence, since most of the reported HTMs have not been tested yet with the mixed-ion perovskites, an objective comparison among different HTMs is not currently possible.

### 3.1. Organic Hole-Transporting Materials

Organic hole-transporting materials can be classified into small-molecule-based, polymer-based and organometallic-complex-based HTMs. We present an overview of the most recent and promising works in view of attaining efficient, inexpensive and stable PSCs. Since the organometallic compounds, like phthalocyanines, still perform relatively poorly compared to other organic HTMs in PSC, we will not describe these systems in the present work. The interested reader can refer to, e.g., [5,52,53].

#### 3.1.1. Small-Molecule-Based HTMs

This category comprises the largest number of newly-designed HTMs for PSC research. Small molecules are suitable when thinking of PSC technology upscaling, because they have a distinct structure and molecular weight and, thus, can be easily reproduced for industrial production with high purity and high yield [39]. Moreover, small organic molecules are superior to conjugated polymers from the viewpoints of the easier synthesis process, better reproducibility, relatively easy tuning of the optical and electrochemical properties by changing different functionalities, low molecular weight and solution processability. Most small-molecule-based HTMs contain nitrogen and sulfur, which are electron-rich atoms and, thus, particularly suitable for HTMs. They are usually based on a triphenylamine (TPA) moiety, due to the presence of the electron-rich nitrogen atom, which minimizes the intermolecular distance, leading to non-planarity of the TPA system. This ultimately results in the formation of amorphous materials, a beneficial feature for HTMs because of their capability to form smooth and pinhole-free films. This ensures uniform contact at the interface with the metal electrodes [54]. However, as mentioned earlier, the amorphous nature of these materials imparts poor hole-mobility. Such a drawback can be overcome by using different organic and hybrid dopants (Figure 4). Nevertheless, one issue regarding the use of dopants is their hygroscopic nature, which reduces their stability and ultimately affects the degradation of the PSCs. The discovery of new stable dopants is an open issue for molecular designers, the discussion of which goes beyond the scope of this article.

Herein, we classify the different small-molecule-based HTMs based on their chemical composition and give a summary of the photovoltaic performance of the presented HTMs (Table 1) as compared to a reference cell containing spiro-OMeTAD.

(a) Pyrene-based HTMs

**Figure 5:**
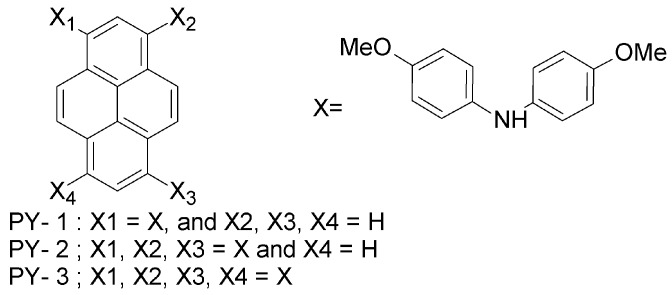
Structures of pyrene-based HTMs: PY-1, PY-2, PY-3.

One of the first works on alternative HTMs appeared in 2013, when Seok et al. proposed a set of pyrene-core arylamine derivative HTMs, with a performance comparable to that of spiro-OMeTAD [55]. In these molecules, the spirobifluorene core of spiro-OMeTAD is replaced by a pyrene core (PY-1, PY-2 and PY-3; Figure 5). Methoxy (-OCH_3_) groups are present also in these pyrene-based HTMs, though their position is changed from *para* (as in each of the quadrants of spiro-OMeTAD) to *meta* or *ortho*. In fact, the -OCH_3_ groups play an important role not only in controlling the electronic properties of spiro-OMeTAD by adjusting the HOMO levels of the materials, but they are also responsible for anchoring the material onto the underlying perovskite layer [4,5]. The electron-donating effect of methoxy groups in the *N*,*N*-di-p-methoxy phenyl amine (X in Figure 5), which is directly bonded to the pyrene moiety, increases the electron density. By enhancing the electron density, both the HOMO and the lowest unoccupied molecular orbital (LUMO) energy levels (and thus, the band gap) are modified. As a result, a high PCE of 12.4% (PY-3) is achieved. PY-1 exhibited lower efficiency (3.3%), which is due to the insufficient driving force for hole injection. The well-known spiro-OMeTAD showed PCE of 12.7% under similar fabrication conditions. The slightly higher PCE of spiro-OMeTAD cells as compared to PY-3 ones originates from the higher short-circuit current (J_SC_), i.e., 21 mA cm^−2^ vs. 20.2 mA cm^−2^, achieved for PY-3 cells, and the higher open-circuit voltage (V_OC_), i.e., 1.01 V (spiro-OMeTAD) vs. 0.93 V (PY-3). This indicates more efficient charge collection via spiro-OMeTAD HTM and better matching between the quasi-Fermi level of the electrons in TiO_2_ and the HOMO of spiro-OMeTAD. Conversely, a higher fill factor is obtained for PY-3 devices with respect to the reference, i.e., 69.5% (PY-3) vs. 59.5% (spiro-OMeTAD). The fill factor is related to the series resistance and shunt resistance, and the enhanced hole-transporting and electron-blocking abilities of PY-3 HTM are responsible for decreased recombination for the photogenerated charges and, thus, higher fill factor. In fact, the introduction of the pyrene core in arylamine derivatives results in HTMs with an electron-blocking ability superior to that of spiro-OMeTAD, while at the same time keeping the synthesis costs lower [55].

(b) Truxene-core HTMs

**Figure 6:**
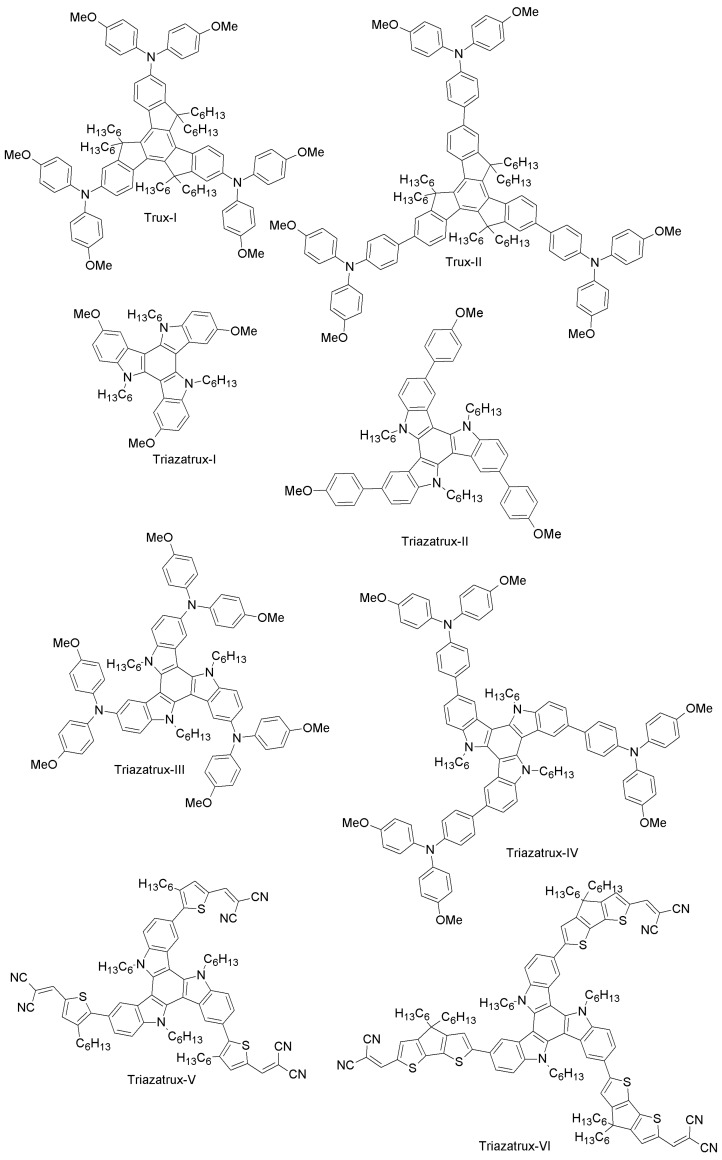
HTMs based on a truxene (Trux) and a triazatruxene (Triazatrux) core.

While most of the papers dealing with alternative HTMs to spiro-OMeTAD assume the use of dopants (as for spiro-OMeTAD itself) to achieve competitive efficiencies, Chen et al. have designed a *C*_3*h*_ truxene-core (Trux-I) with OMeTAD terminals and hexyl side-chains (Figure 6) [57]. Its planar, rigid and fully-conjugated structure results in an excellent hole-mobility of the pristine material of roughly 10^−3^ cm^2^V^−1^s^−1^, nearly two orders of magnitude higher than that of spiro-OMeTAD and polytriarylamine (between 10^−5^ and 10^−4^ cm^2^V^−1^s^−1^ [98]). Indeed, its higher mobility allows one to fabricate devices with a superb PCE of 18.6%, without introducing any external dopants [57].

Recently, Grisorio et al. have synthesized Trux-I and a new molecule named Trux-II (Figure 6) [56]. These star-shaped HTMs were designed by binding the bis(p-methoxyphenyl)amine groups to a truxene-based core (Trux-I) and by interspacing these electron-donating functionalities from the core with 1,4-phenylene π-bridges (Trux-II). The authors have subsequently employed them as HTMs in two different perovskite device architectures (direct and inverted). As for the inverted configuration (n-i-p), both HTMs showed a poor performance (PCE = 4.9% and 5% for Trux-I and Trux-II, respectively) with respect to spiro-OMeTAD (19.2%). However, in the case of direct device configuration (p-i-n), the trend was dramatically different: both Trux-I- and Trux-II-containing cells outperformed the spiro-OMeTAD reference (PCE = 10.2%, 13.4%, 9.5% for Trux-I, Trux-II and spiro-OMeTAD, respectively) [56]. The huge difference in the photovoltaic behavior achieved in the two configurations depends on the intramolecular charge distributions in radical cations and on the thickness of the HTMs (5–20 nm and 150–200 nm in inverted and direct configuration, respectively). This study indicates that the performance of PSCs can be effectively tuned by ad hoc device architecture modifications.

Rakstys et al. have designed and synthesized a series of four two-dimensional triazatruxene-based derivatives (Triazatrux-I, Triazatrux-II, Triazatrux-III and Triazatrux-IV; Figure 6) using inexpensive starting materials and simple synthetic procedures for low production costs [58]. These centrosymmetric star-shaped HTMs, which comprise a planar triazatruxene core and electron-rich methoxy-engineered side arms, interact efficiently with the perovskite surface (a mixed perovskite composition, (FAPbI_3_)_0.85_(MAPbBr_3_)_0.15_, was chosen), thus providing better hole-injection from perovskite to HOMO levels of the HTMs, as revealed by the time-resolved photoluminescence studies. The Triazatrux-II-based solar cell exhibited power conversion efficiency of 17.7%, which is slightly higher than that of spiro-OMeTAD device (17.1%) [58].

The triazatruxene-based design guidelines open new paths for constructing low-cost and high-performance hole-transporting materials for PSCs. Rakstys et al. recently designed for the first time a series of star-shaped triazatruxene-based donor-π-acceptor HTMs (Triazatrux-V, Triazatrux-VI and Triazatrux-VII; Figure 6) [59]. When studying their application in PSCs, they observed that Triazatrux-VII led to high PCEs (19%), on par with those of spiro-OMeTAD cells. This exceptionally good performance is attributed to a particular face-on stacking organization of Triazatrux-VII on perovskite (a mixed composition, (FAPbI_3_)_0.85_(MAPbBr_3_)_0.15_, was chosen) films, which favors vertical charge carrier transport through an ordered structure. These results are particularly interesting because they represent a unique example of highly-efficient PSCs based on a pristine HTM without any chemical additives or doping. This work paves the way toward the molecular design of next-generation HTMs with high mobility based on a planar donor core, p-spacer and periphery acceptor [59].

(c) Phenothiazine-based HTMs

**Figure 7:**
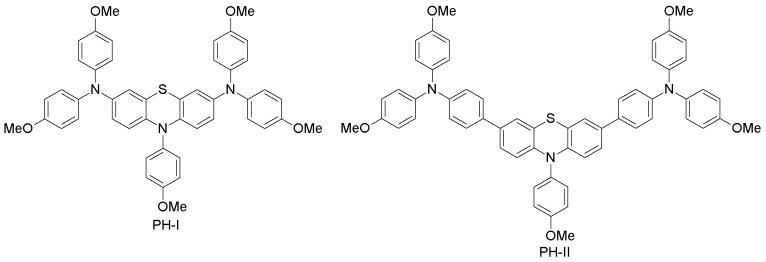
Phenothiazine-based HTMs.

The phenothiazine heterocycle plays an important role in the design of high-mobility organic semiconducting materials [99]. Because of their excellent optical, electrochemical and thermal properties, phenothiazine-based sensitizers have been widely used in DSSCs with great performance [100]. Recently, Grisorio and co-workers designed and synthesized two phenothiazine-based molecules, which differ in the aromatic linker (PH-I and PH-II; Figure 7) [60]. PH-I and PH-II were synthesized through straightforward Buchwald−Hartwig and Suzuki−Miyaura cross-couplings, by binding diarylamine or triarylamine groups to a phenothiazine core, respectively. When used as HTM in PSCs, PH-I led to a poor power conversion efficiency of 2.1%, while on the other hand, PH-II exhibited an exceptional PCE of 17.6%, which is close to that obtained with spiro-OMeTAD HTM (17.7%) under the same conditions [60]. The oxidation potential of PH-II (−5.15 eV, which is close to that of spiro-OMeTAD of −5.02 eV) results in high open-circuit voltage (1.11 V for PH-II cells vs. 0.82 V for PH-I devices). Nevertheless, the lower oxidation of PH-I (−4.77 eV) with respect to perovskite (−5.4 eV) is responsible for the more efficient hole-transfer from perovskite to the HOMO level of PH-I. The significantly different photovoltaic behavior of PH-I and PH-II is attributed to the modulation of the electron density distribution, which affects the stability of the molecules during the charge-transfer dynamics at the perovskite|HTM interface. This study demonstrates that, upon minor modifications to the phenothiazine unit, one can achieve significant changes in the PSC performances by low-cost alternatives to spiro-OMeTAD HTM.

(d) Acridine-, thiophene-, biphenyl-, bithiophene-, tetrathiophene- and phenyl-based HTMs

**Figure 8:**
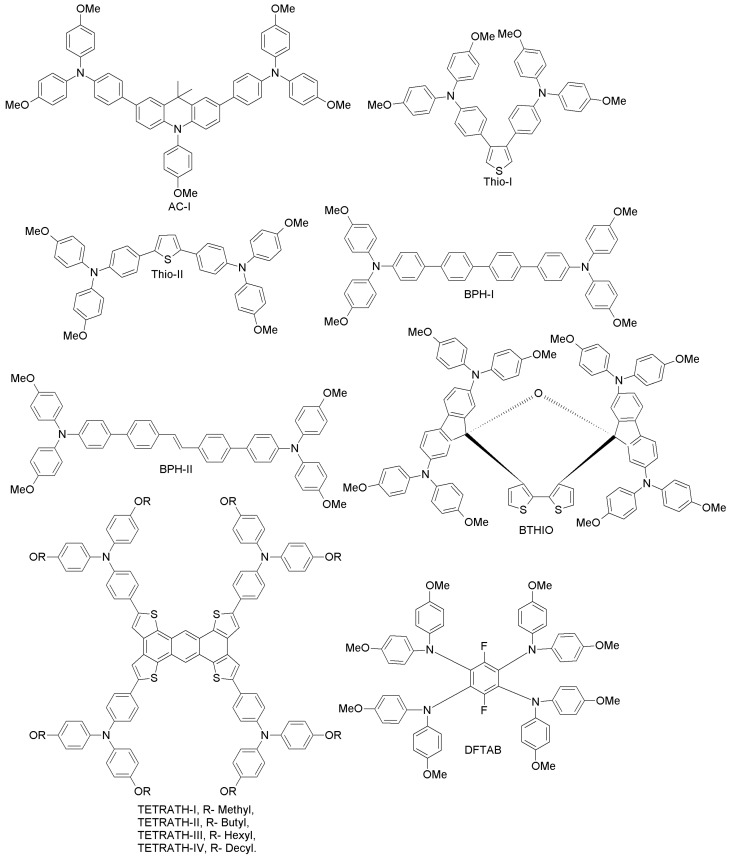
Acridine-, thiophene-, biphenyl-, bithiophene-, tetrathiophene- and phenyl-based HTMs.

Chao et al. have reported an acridine-based hole-transporting material (AC-I; Figure 8) with a 9,9-dimethyl-9,10-dihydroacridine core, prepared by an easy synthetic procedure consisting of two steps and with good reaction yields [61]. In fact, AC-I does not contain the spirobifluorene motif typical of spiro-OMeTAD, whose preparation requires highly intricate synthetic strategies. Its hole-mobility (in the order of 10^−3^ cm^2^V^−1^s^−1^, upon doping of additives such as Li-TFSI and tertiary butyl pyridine) is comparable to that of spiro-OMeTAD, and its HOMO level (−5.03 eV) is slightly lower than that of spiro-OMeTAD (4.97 eV). When AC-I was employed as HTM for a perovskite device, a power conversion efficiency of 16.42%, comparable to that of spiro-OMeTAD under the same conditions (16.26%), was achieved after HTM thickness optimization (~250 nm), due to enhanced charge separation kinetics and recombination resistance. Hence, acridine-based derivatives can be useful low-cost alternatives to spiro-OMeTAD. The synthetic costs of AC-I are estimated to be approximately half of the costs of spiro-OMeTAD. Furthermore, AC-I can be synthesized in larger quantities with a high yield against spiro-OMeTAD [61].

Liu and co-workers have designed two thiophene-substituted HTMs (Thio-I and Thio-II; Figure 8), by a simple one-step synthesis of dibromo thiophene with arylamine [62]. The substitution position of the arylamine moieties on the thiophene π-linker in the two HTMs was connected to the PSC performance via computational and experimental studies. Thio-II showed better hole-mobility than Thio-I, due to its favorable conjugation in the 2,5 positions as compared to that in the 3,4 positions of Thio-I. As a result, a good overall solar cell performance of 15.13% in a Thio-II-based PSC was achieved, which is 40% higher than that obtained with Thio-I-containing HTM. This indicates that favorable geometry of HTMs results in enhanced PSC performance. In the same work, when spiro-OMeTAD was adopted as HTM with a concentration of 20 mg mL^−1^ under similar conditions, a surprisingly poor performance (PCE = 8.83%) was achieved. However, when the spiro-OMeTAD concentration was increased up to 73 mg mL^−1^, PCE was enhanced up to 15.63%.

Pham et al. prepared two easily-attainable, biphenyl-based, low-cost and high-performance HTMs for PSCs (BPH-I, BPH-II; Figure 8) via conventional Suzuki coupling reactions [63]. In particular, BPH-II-based cells exhibited a PCE of 16.42% (spiro-OMeTAD employed under similar conditions led to PCE of 16.81%), suggesting that BPH-II could be a good low-cost replacement for spiro-OMeTAD. Regarding the device stability, PSCs based on BPH-I and BPH-II retain almost 87% of the initial performance after 10 days, similar to spiro-OMeTAD devices.

Rakstys et al. developed a bithiophene-based derivative (2,2’,7,7’-tetrakis-(*N*,*N*’-di-4-methoxyphenylamine)dispiro-[fluorene-9,40-dithieno[3,2-c:20,30-e]oxepine-6’,9’’-fluorene], BTHIO; Figure 8) and studied its performance, stability and crystallography [64]. BTHIO, a novel dispiro-oxepine derivative, was prepared by using a simple three-step synthetic procedure and low-cost precursors. When adopted as HTM, the corresponding PSC exhibited one of the best reported power conversion efficiencies of 19.4%, slightly higher than that of the spiro-OMeTAD reference cell (18.8%) under similar conditions. Furthermore, BTHIO shows significantly improved stability when compared to spiro-OMeTAD-based cells.

In a very recent report, Zimmermann et al. designed highly electron-rich tetrathiophene-fused HTMs (TETRATH-I–IV; Figure 8), differing from each other with respect to the alkoxy groups (methyl, butyl, hexyl and decyl, respectively) [65]. All of these derivatives are easy to synthesize and to purify. Moreover, TETRATH-I showed higher thermal stability and performance (PCE = 18.1%), comparable to the conventional spiro-derivative. Upon introduction of different alkyl groups, the solubility of the tetrathiophene core increased, but at the same time, the efficiency decreased dramatically up to 9.7% (TETRATH-IV). In case of TETRATH-I, the solubility increased by heating to 100 °C prior to spin coating. The PSC performance remained at a high level after heating, yet when a similar experiment was conducted for the traditional spiro-derivative, the PCE decreased dramatically already upon heating to 70 °C. This indicates that tetrathiophene can enable the design of thermally-stable and low-cost PSCs with high performance [65].

Chen et al. have reported a simple HTM (3,6-difluoro-N1,N1,N2,N2,N4,N4,N5,N5-octakis(4-methoxyphenyl)benzene-1,2,4,5-tetraamine, DFTAB; Figure 8), obtained via one-step synthesis using commercially available precursors [66]. When utilized in a PSC, it gave rise to a PCE of 10.4% with low hysteresis. When DFTAB was used without additional ionic dopants, the corresponding device achieved a stabilized PCE of 6%. The low cost and easy synthesis render this HTM promising for future large-scale applications, especially considering that avoiding additional dopants will significantly enhance the long-term stability of the PSCs.

(e) Triazine-based HTMs

**Figure 9:**
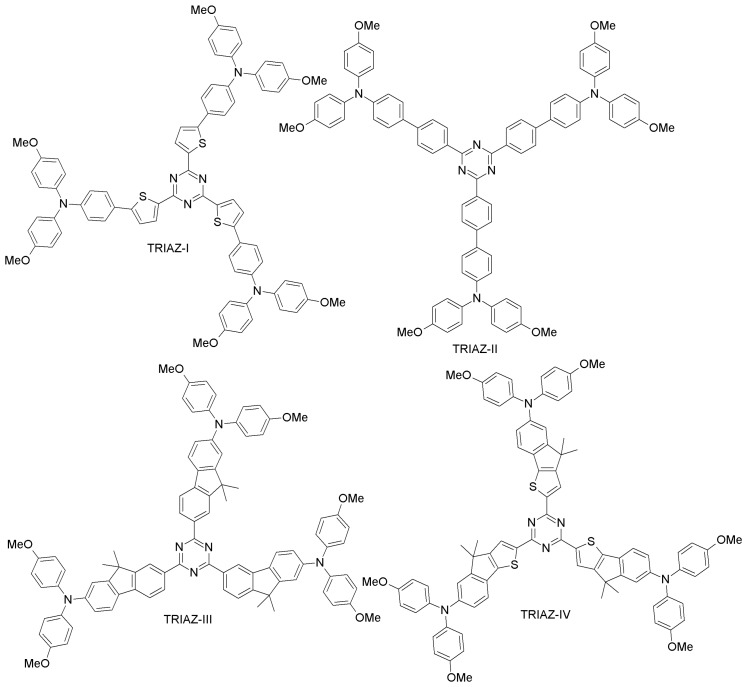
Triazine-based HTMs.

**Figure 10:**
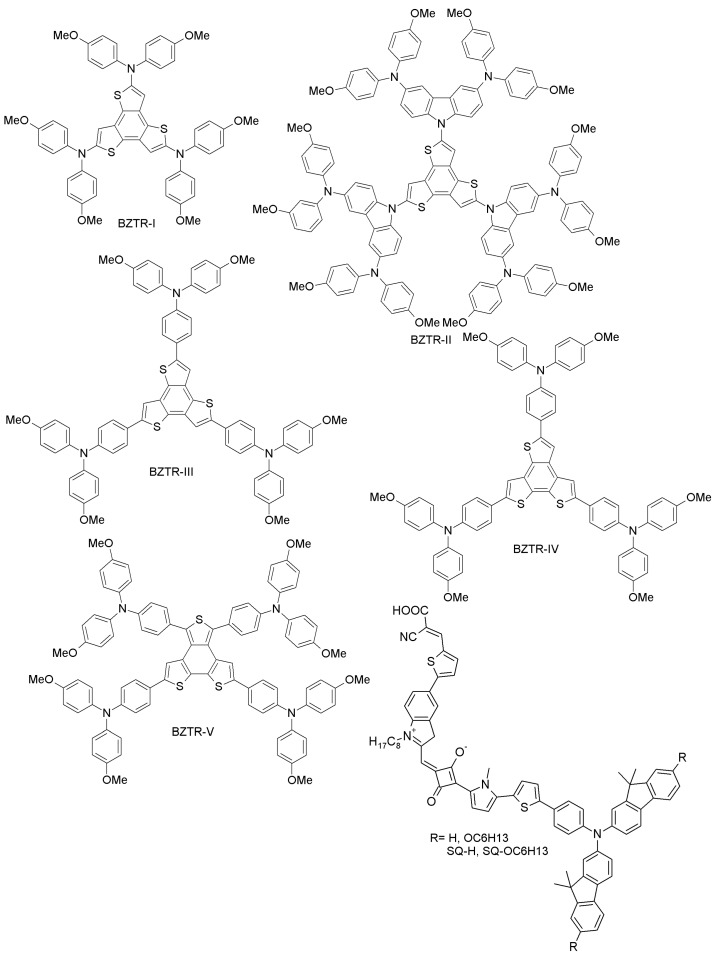
Benzotrithiophene (BZTR)- and squaraine (SQ)-based HTMs.

Ko and co-workers synthesized electron-deficient triazine core donor-acceptor HTMs (TRIAZ-I and TRIAZ-II; Figure 9), differing by the spacer group (thiophene or phenyl group) and with dimethoxytriphenylamine as the donor moiety [67]. Both TRIAZ-I and TRIAZ-II showed hole-mobility similar to that of spiro-OMeTAD. When employed as HTMs in PSCs, TRIAZ-I exhibited better performance than TRIAZ-II (12.5% and 10.90% efficiencies, respectively) due to a higher photocurrent and fill factor. Under similar conditions, spiro-OMeTAD-based PSC showed a PCE of 13.45%. Lim et al. presented two triazine core star-shaped HTMs (TRIAZ-III and TRIAZ-IV; Figure 10) [68]. They found that TRIAZ-IV exhibited a red-shift in the absorption band, as well as better hole-mobility as compared to TRIAZ-III, due to the presence of the electron-rich indeno[1,2-b]thio moiety. These two materials led to excellent PCEs (13.2% and 12.6% for TRIAZ-III and TRIAZ-IV, respectively), comparable to spiro-OMeTAD (13.8%).

(f) Benzotrithiophene- and squaraine-based HTMs

Ontoria et al. have obtained three benzotrithiophene-based HTMs (BZTR-I, BZTR-II and BZTR-III; Figure 10) by straightforward cross-coupling reactions between different triphenylamine derivatives and benzotrithiophene [69]. These materials, when further applied to PSCs, showed PCEs of 16%, 17, and 18.2% for BZTR-I, BZTR-II and BZTR-III, respectively, comparable to the reference spiro-OMeTAD (PCE = 18.1%) under similar conditions. The higher performance of BZTR-III is due to its better conductivity and good alignment of the HOMO level to the perovskite valence band. Along the same line, Benito et al. have prepared tri-arm and tetra-arm isomers (BZTR-IV and BZTR-V; Figure 10) and studied their optical, electrochemical, photophysical properties and PSC performance [70]. These materials are highly stable up to 430 °C, and the corresponding photovoltaic devices show superior performance with PCE of 19% for BZTR-IV and 18.2% for BZTR-V. The higher efficiency achieved with the derivative BZTR-IV may be related to its *cis*-sulfur arrangement, leading to favorable interactions with the perovskite structure and better hole extraction. Paek et al. have recently designed squaraine-based (SQ-H, SQ-OC_6_H_13_; Figure 10) HTMs, identifying them as excellent light harvesters in PSCs [71]. These HTMs exhibited excellent PCEs of 14.74% (SQ-H) and 14.73% (SQ-OC_6_H_13_), comparable to the spiro-OMeTAD reference (PCE 15.33%). The air stability of these materials was also investigated: for SQ-H, the PCE dropped only by 12% upon 300 h of ambient exposure, while for SQ-OC_6_H_13_, there was no change in PCE.

(g) Fluorene- and spiro-fluorene-based HTMs

**Figure 11:**
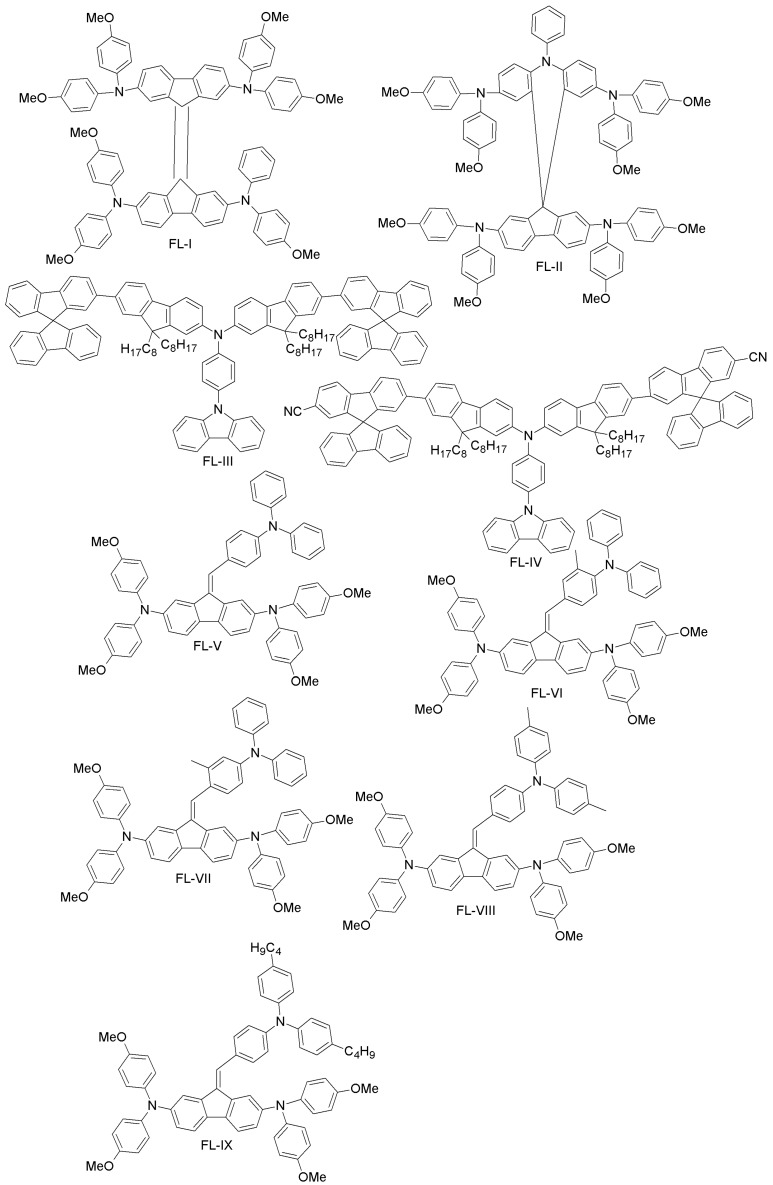
Fluorene-based HTMs for high-performance PSCs.

Rakstys et al. designed a novel bifluorenylidene-based HTM (FL-I; Figure 11) with a lower band-gap (2.41 eV) compared to spiro-OMeTAD (3.00 eV), yet with a similar HOMO level (−5.09 eV vs. −5.04 eV) [72]. When FL-I was employed as an HTM in PSC, it exhibited a power conversion efficiency of 17.8%, comparable to spiro-OMeTAD (18.4%). However, being almost 50-times less expensive than spiro-OMeTAD, FL-I is an intriguing candidate for future commercial applications of PSCs.

As already mentioned, various dopants have been used in HTMs in order to enhance their electrical conductivity, while at the same time decreasing the stability and increasing the cost of the resulting device. Based on these considerations, Wang et al. designed a dopant-free HTM (FL-II; Figure 11), which constituted both the polytriarylamine unit (i.e., N-benzene) and spiro-OMeTAD [73]. FL-II showed excellent PCE of 16.73% with dopants and 12.39% without dopants. The corresponding values for a spiro-OMeTAD-based device are 14.84% and 5.91%, respectively.

Focusing on device stability, Reddy and co-workers developed two fluorene-based HTMs (FL-III and FL-IV; Figure 11) using the Suzuki coupling reaction. In their molecular design, spiro-fluorene was end-capped to a terminal fluorine group of two different sides, while carbazole was linked to the third arm of the triphenylamine core [74]. The two materials differ through the cyano-group end-capping. For both, the HOMO levels were well aligned with spiro-OMeTAD and PEDOT:PSS. In addition, both exhibited high hole-mobility, long-term stability and good solubility. Due to such attractive characteristics, both have been employed in PSCs (replacing spiro-OMeTAD), as well as in the organic bulk-heterojunction (BHJ; replacing PEDOT:PSS). The reported PCE for FL-III was 17.25%, higher than for the spiro-OMeTAD reference (16.67%). In a BHJ cell, a PCE of 7.93% was achieved, the reference PEDOT:PSS cell yielding an efficiency of 7.74%.

Tiazkis et al. have systematically studied the structure-property relationship of a series of fluorene-based HTMs (FL-V, FL-VI, FL-VII, FL-VIII and FL-IX; Figure 11) [75]. They found that hole extraction, molecular planarity and charge-transport properties can be tuned by substitution of an aliphatic group to the *meta-* and *para*-positions of triphenylamine fragments. The poor performance observed in the case of *meta*-substitution may be due to the non-favorable geometry, but in the case of *para*-substitution, the overall PCE fell in the 9%–16.8% range, close to the spiro-OMeTAD reference value (17.8%).

**Figure 12:**
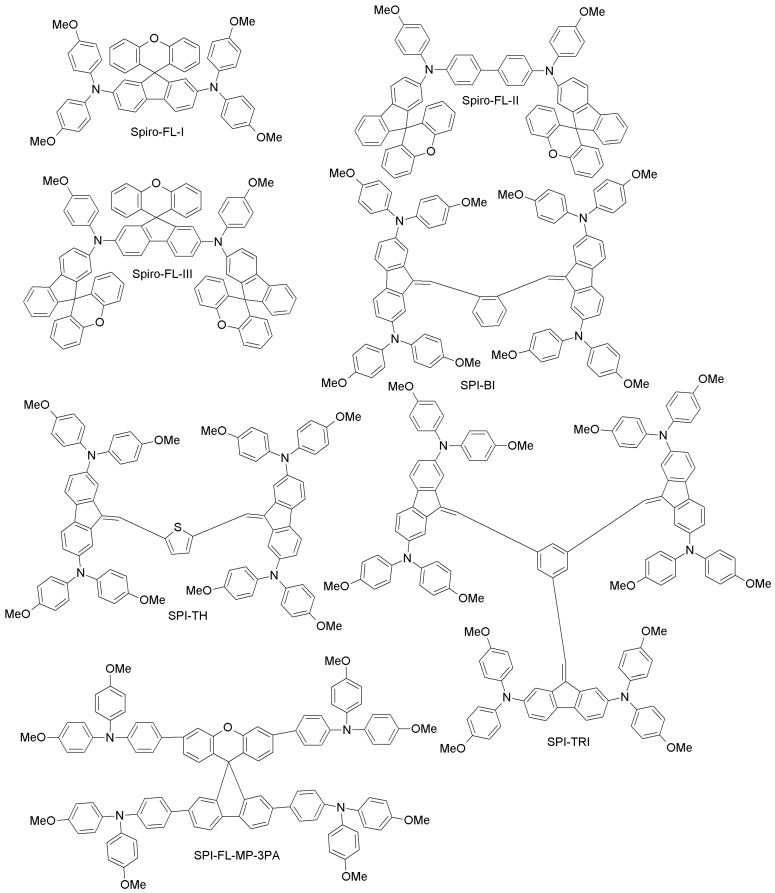
Spiro-fluorene-based HTMs (1).

Since spiro-OMeTAD-based derivatives are found in many of the best-performing HTMs, Bi and co-workers recently reported about a newly-designed and easily-synthesized spiro-based HTM (spiro-FL-I; Figure 12) with excellent performance (PCE = 19.8%; 20.8% for the spiro-OMeTAD reference) [76]. In addition, devices based on spiro-FL-I showed less hysteresis, excellent reproducibility in device fabrication and better stability under dark and dry conditions. By following a similar design principle, Xu et al. have prepared two 3D-spiro-fluorene-based HTMs (spiro-FL-II, spiro-FL-III; Figure 12) [77]. Spiro-FL-III exhibited better hole-mobility, better film-forming properties, higher solubility and a deeper HOMO level as compared to spiro-OMeTAD, also yielding higher PCE (20.8% vs. 18.8%; the PCE for spiro-FL-II was 13.6%). Spiro-FL-III showed also excellent stability after long-term aging for six months. Malinauskas and co-workers have reported a set of HTMs with di-substituted and tri-substituted benzene, or a di-substituted thiophene core (SPI-BI, SPI-TH and SPI-TRI, respectively; Figure 12) with fluorine as peripheral unit [79]. The hole-mobility of these materials is in the same order as for spiro-OMeTAD, but their advantage is that they are easy to prepare via two steps, using commercially available precursors, with an overall material cost equal to about one fifth that of spiro-OMeTAD. When employed in PSCs, these HTMs exhibited PCE values up to 20%. Liu et al. have recently reported a series of four spiro-fluorene derivatives differing from each other by the *para*- and *meta*-substitution of diphenylamine or triphenylamine moieties (SPI-FL-MM-3PA, SPI-FL-MP-3PA, SPI-FL-MM-2PA and SPI-FL-MP-2PA; Figure 12) [80]. Furthermore, these compounds showed high hole-mobility, good solubility, suitable energy levels and efficient hole-extraction combined with electron-blocking capability, which are all positive characteristics of HTMs. In the devices, SPI-FL-MP-2PA exhibited the best photovoltaic performance, with a PCE of 16.8%, slightly higher than that of the reference spiro-OMeTAD (15.5%) under similar conditions [80]. When SPI-FL-MP-2PA was further adopted in mixed FAPbI_3_/MAPbBr_3_ PSCs, an enhanced PCE of 17.7%, comparable to the spiro-OMeTAD reference (17.6%), was achieved. Furthermore, SPI-FL-MP-2PA-based devices possess drastically improved stability compared to spiro-OMeTAD-containing devices, with 90% of initial PCE retained after 2000 h in ambient temperature, while spiro-based devices retained only 20% of initial PCEs under the same time exposure.

To overcome the stability drawbacks of HTM additives, Xu and co-workers have designed dopant-free HTMs based on spiro-fluorene derivatives (one-3′,6′-bis(benzyloxy)-N2,N2,N7,N7-tetrakis(4-methoxyphenyl)spiro [fluorene-9,9′-xanthene]-2,7-diamine (XDB), N2,N2,N7,N7-tetrakis(4-methoxyphenyl)-3′,6′-bis(pyridin-2-ylmethoxy)spiro[fluorene-9,9′-xanthene]-2,7-diamine (XOP), N2,N2,N7,N7-tetrakis(4-methoxyphenyl)-3′,6′-bis(pyridin-3-ylmethoxy)spiro[fluorene-9,9′-xanthene]-2,7-diamine (XMP), and N2,N2,N7,N7-tetrakis(4-methoxyphenyl)-3′,6′-bis(pyridin-4-ylmethoxy)spiro[fluorene-9,9′-xanthene]-2,7-diamine (XPP); Figure 12), in which the pyridine group is the pendant to respective HTMs with different positions of nitrogen atoms (*para*, *ortho* and *meta* substitution) [78]. Among these, XPP exhibited a PCE of 17.2% without the use of external dopants, which is much higher than corresponding PCE for the spiro-OMeTAD cell (5.5%) under similar conditions. When planar PSCs were considered, XPP-based HTMs even yielded a PCE of 19.5% without any external dopant. Further studies of steady-state PL and transient photocurrent decays for these materials revealed much better hole-extracting and hole-transporting capabilities than for spiro-OMeTAD, thus explaining the better performance and long-term stability as compared to the conventional HTM.

**Figure 13:**
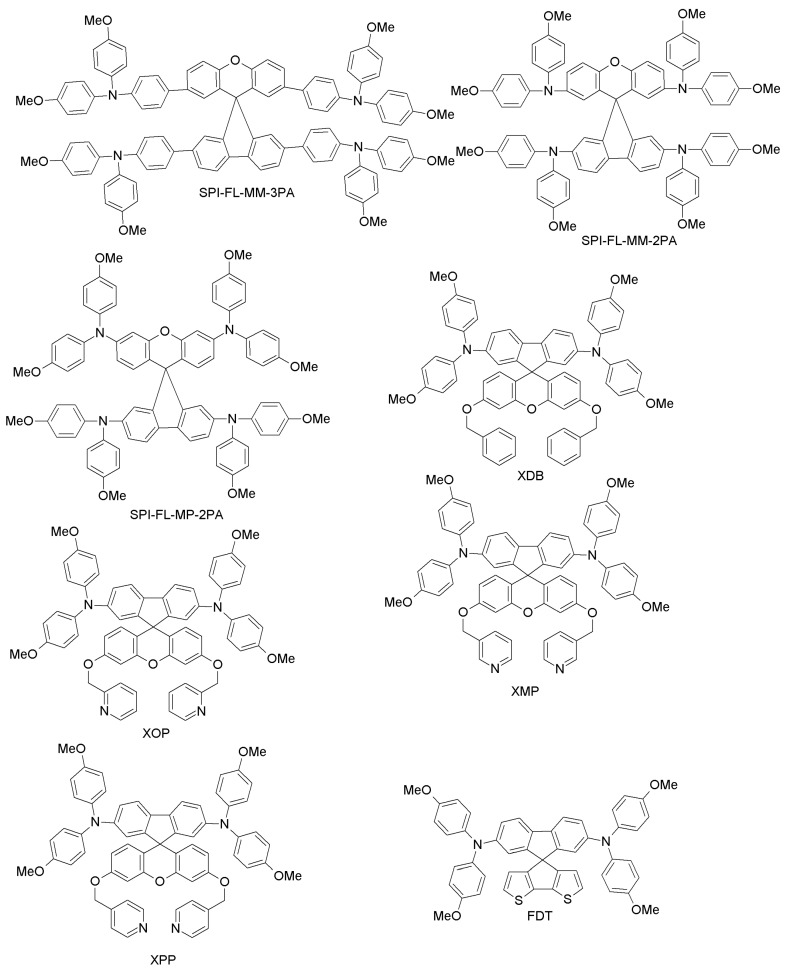
Spiro-fluorene-based HTMs (2).

One of the most interesting examples of organic HTMs is the one recently proposed by Saliba et al. [39]. They present a novel compound, 2’,7’-bis(bis(4-methoxyphenyl)amino)spiro[cyclopenta[2,1-b:3,4-b’]dithiophene-4,9’-fluorene] (FDT; Figure 13), where an asymmetric fluorine-dithiophene core is substituted by *N,N*-di-p-methoxyphenylamine donor groups. FDT was designed on the basis of the interesting optoelectronic properties of spiro-cyclopentadithiophene derivatives. When used in a mesoscopic configuration instead of spiro-OMeTAD, it leads to one of the highest PCEs reported for small-molecule HTMs, i.e., 20.2%. The advantages of FDT are the low costs of the material (~60 $/g), and the possibility of using toluene for dissolving it, instead of the more hazardous chlorobenzene used for spiro-OMeTAD [39]. These results are particularly interesting because they point towards an entire class of new HTMs with high performance by molecular engineering of the FDT core.

(h) Carbazole-based HTMs

Carbazole-based derivatives have attracted much attention as charge-transporting materials for organic light-emitting diodes (OLEDs), DSSCs and PSCs [101,102,103]. Their interesting photophysical properties such as intense luminescence and reversible oxidation processes, together with the reasonable synthetic costs, the versatility of the carbazole reactive sites and the excellent charge transport properties justify the efforts to find novel solutions for low-cost HTMs for PSCs in this class of compounds [10,40,82,83,84,86,87,88,89,90,91,92].

**Figure 14:**
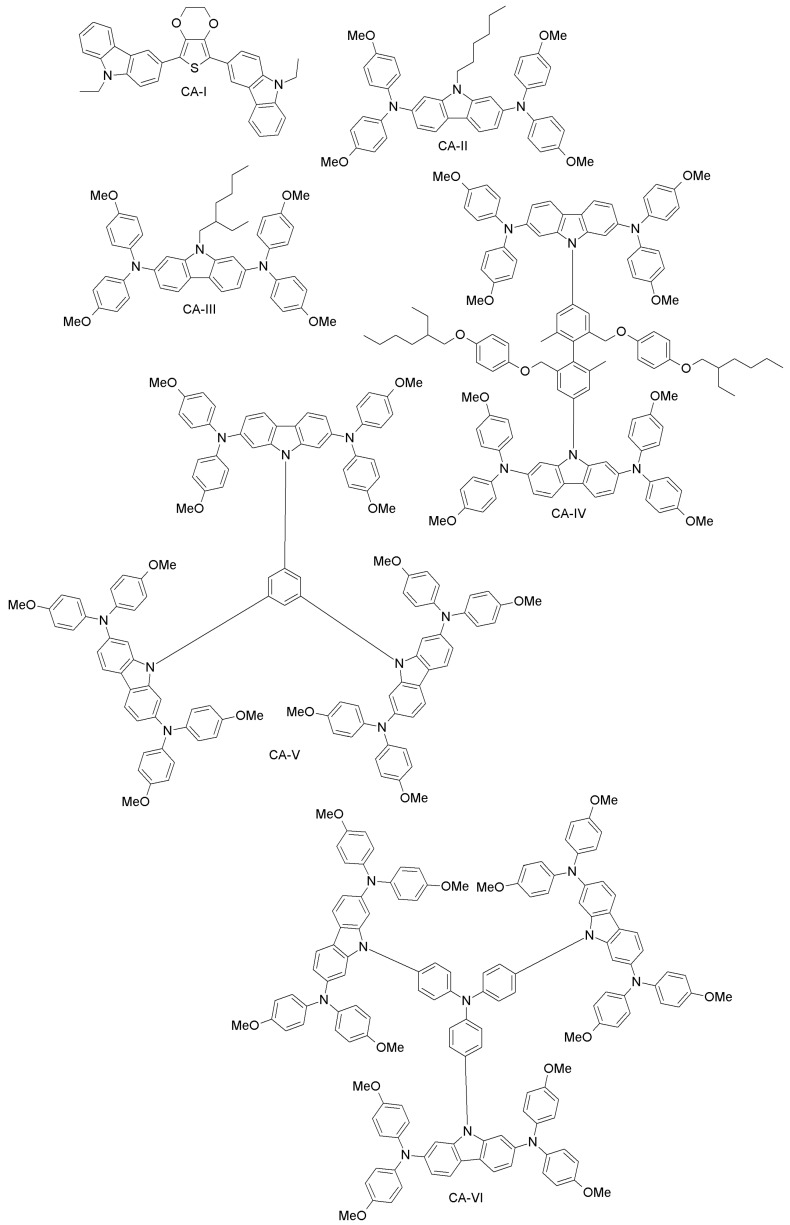
Carbazole-based HTMs (1).

A study from Wu et al. reports a carbazole-based compound (CA-I; Figure 14) synthesized with a simple two-step reaction from inexpensive and commercially available materials [81]. CA-I has higher hole-mobility and conductivity than spiro-OMeTAD, and it leads to 12.3% efficiency for PSCs, which is comparable to what the authors achieved with spiro-OMeTAD [104].

**Figure 15:**
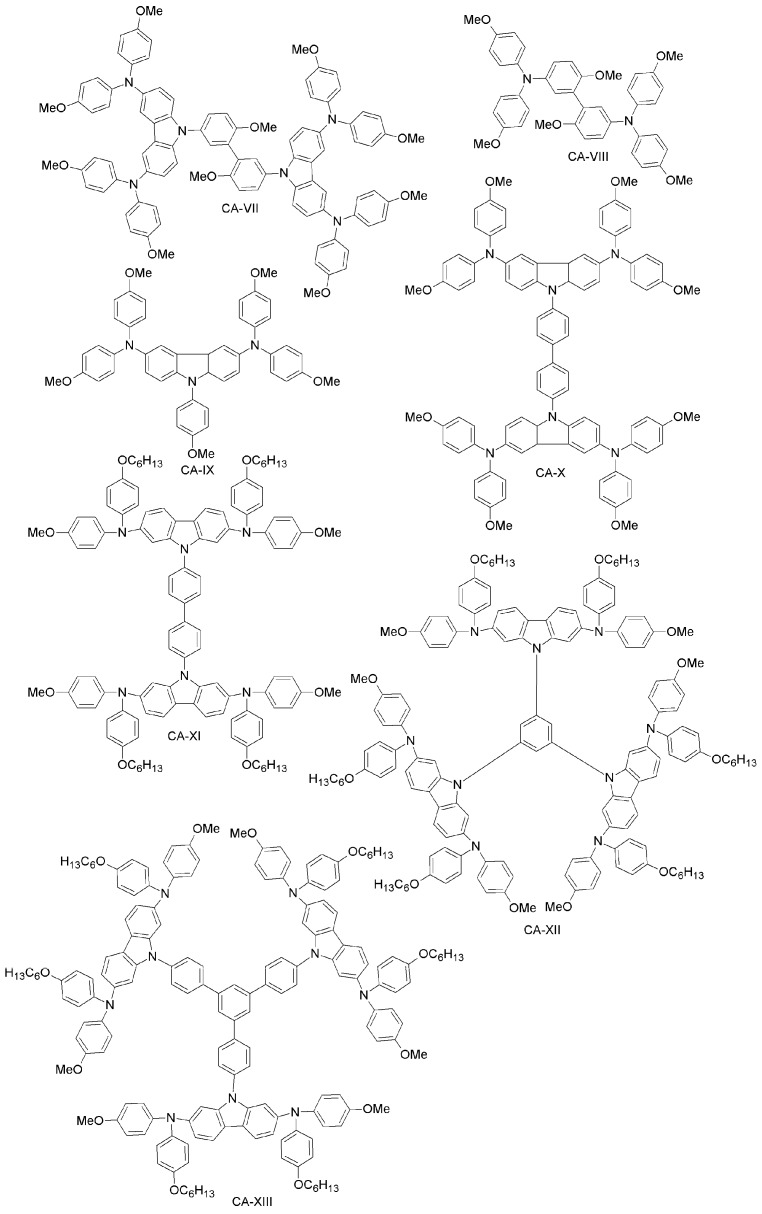
Carbazole-based HTMs (2).

Leijtens et al. have synthesized two simple carbazole-based, lithium salt-free HTMs, differing by the alkyl group (linear or branched; CA-II and CA-III, respectively; Figure 14) [82]. The oxidized form of these materials, when applied in PSCs, produced similar performance to that of spiro-OMeTAD-based doped devices, thus implying that simple carbazole-based design can be useful for making dopant-free PSCs. Another example is a carbazole-based HTMs with two-arm and three-arm structures, connected through TPA, phenylene or diphenylene core units (CA-IV, CA-V, CA-VI; Figure 14) [83]. With these systems, a PCE as high as 14.79% was obtained. Recently, Wang et al. synthesized novel carbazole-based derivatives (CA-VII, CA-VIII; Figure 15) with a biphenyl core. Undoped CA-VII resulted in a PCE of 4.53%, close to that of spiro-OMeTAD (5.10%). CA-VIII, in turn, showed much poorer efficiency of 0.19%, due to the mismatched HOMO level with respect to the perovskite and increased electron recombination at the interface [84].

**Figure 16:**
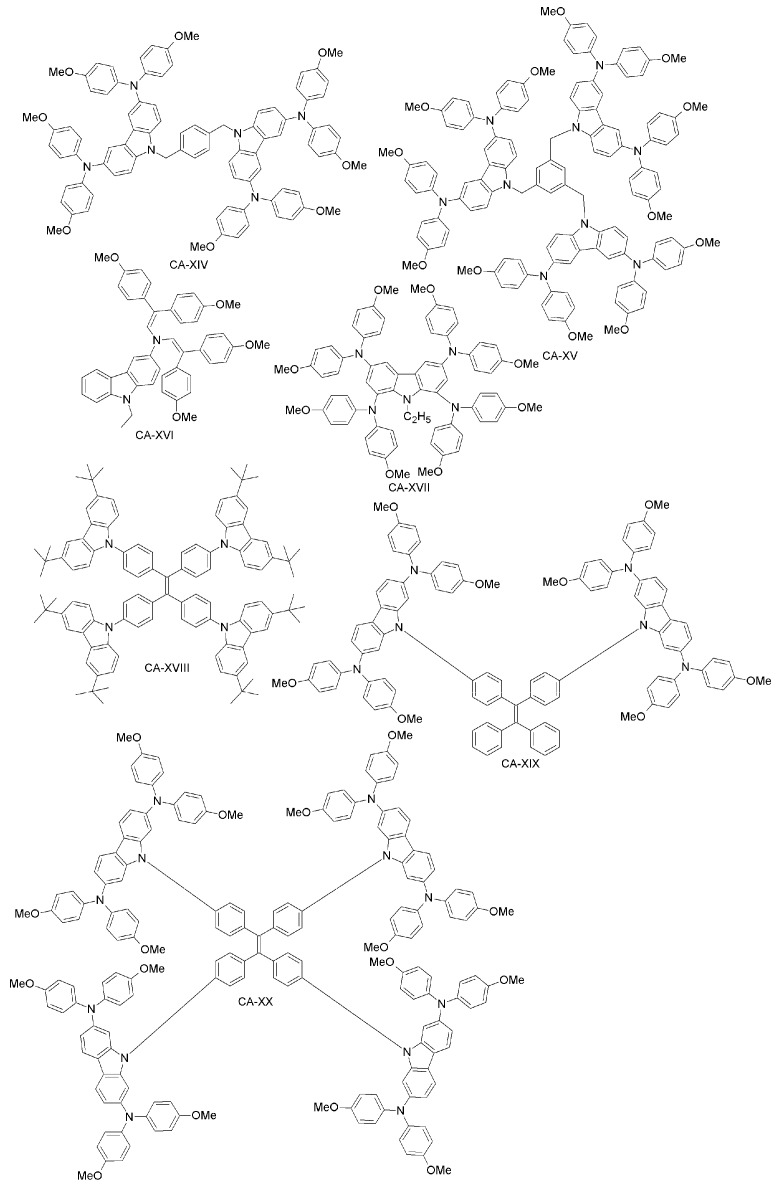
Carbazole-based HTMs (3).

Xu et al. have reported two HTMs (CA-IX, CA-X; Figure 15) [92] based on the carbazole-core. These materials had previously been used in solid-state DSSCs using (E)-3-(6-(4-(Bis(2’,4’-dibutoxy-[1,1’-biphenyl]-4-yl)amino)phenyl)-4,4-dihexyl-4H-cyclopenta[2,1-b:3,4-b’]dithiophen-2-yl)-2-cyanoacrylic Acid (LEG4) as a sensitizer [85]. CA-X cells exhibited higher PCE (6%) than those employing CA-IX (4.5%), while under the same conditions, the performance of the spiro-OMeTAD-based device was 5%. The good performance of CA-X in solid-state DSSCs gives strong evidence of its superiority over spiro-OMeTAD, pointing towards its further use as HTM in PSCs. The CA-X-based PSC device exhibited a power conversion efficiency of 9.8% (spiro-OMeTAD reference: 10.2%). The superiority of CA-X as compared to CA-IX might be due to lower reorganization energy, yielding higher hole-mobility than in CA-IX. Kang et al. have designed a family of dendritic carbazole-based star-shaped HTMs (CA-XI, CA-XII, CA-XIII; Figure 16) and investigated their performance in PSCs [86]. The trimeric structures exhibited higher conductivity than the dimeric one, due to crystallization during the fabrication process. As HTM, CA-XIII resulted in a PSC with 13% efficiency, which is comparable to that of spiro-OMeTAD PSCs (13.76%). Recently, Zhang et al. synthesized two new carbazole-based materials (CA-XIV and CA-XV; Figure 16) using commercially available di-substituted and tri-substituted phenyl derivatives [87]. The PSCs based on these HTMs had PCEs of 11.4% and 13.1% for CA-XIV and CA-XV, respectively (spiro-OMeTAD reference: 12.0%).

**Figure 17:**
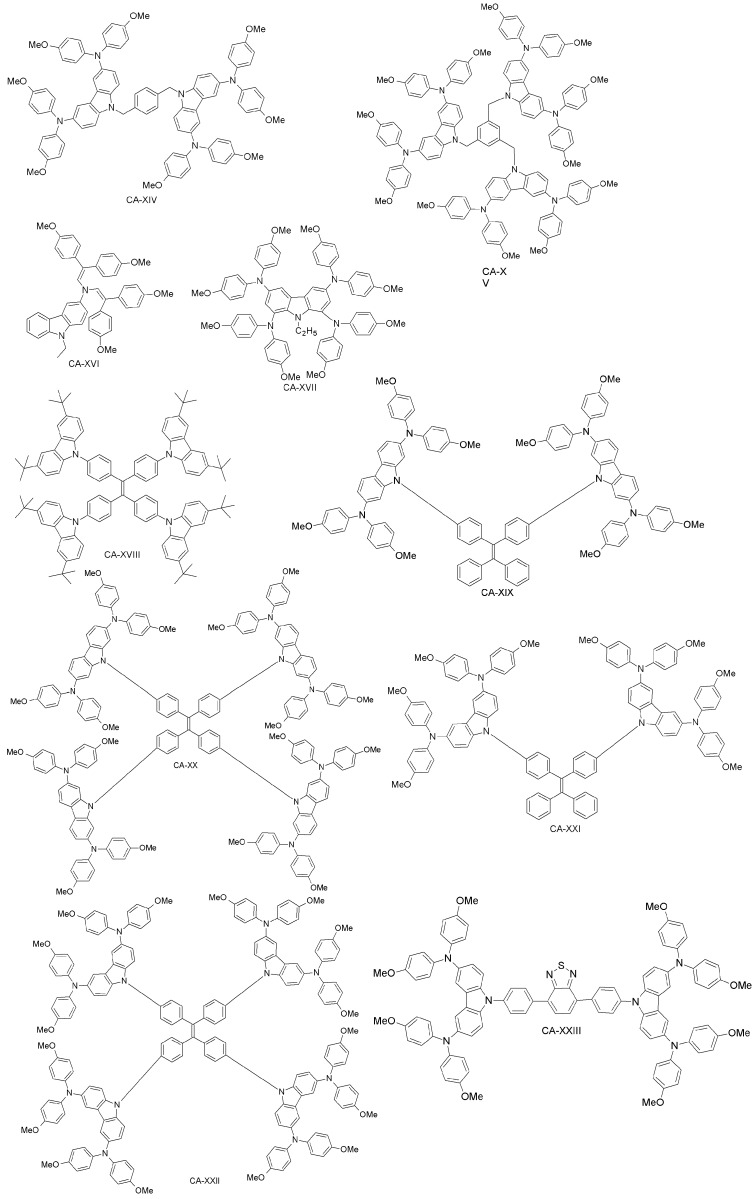
Carbazole-based HTMs (4).

Daskeviciene et al. have also developed a simple one-step synthesis for obtaining a low-cost HTM (CA-XVI; Figure 16) [88]. Such an HTM led to PSCs with 17.8% efficiency, comparable to the spiro-OMeTAD reference device. Simple enamine condensation chemistry for synthesis, low-cost materials and higher efficiency suggest the applicability of CA-XVI designs as HTMs for future high-performance and low-cost organic electronic devices. Chen et al. reported a tetra-substituted carbazole-based HTM (CA-XVII; Figure 17) via a three-step synthesis, using low-cost starting materials, with a PCE of 17.81% [89]. The authors also investigated the role of the OMeTAD group in the system, by synthesizing a molecule similar to CA-XVII, but with a different substituent than OMeTAD. The corresponding photovoltaic device had extremely low performance (PCE close to zero), which demonstrates the importance of the OMeTAD moiety in the HTM design. Zhu and co-workers have prepared a carbazole-based molecular design with a tetra-phenylene core (CA-XVIII; Figure 16) [90]. CA-XVIII imparts good thermal stability and well-aligned energy levels. When used as HTM, the resulting PSCs exhibited a PCE of 12.4% without dopants, which is close to 14.3% obtained with doped spiro-OMeTAD. Recently, Zhu et al. synthesized a series of carbazole derivatives differing in the 2,7 and 3,6 positions (CA-XIX, CA-XX, CA-XXI and CA-XXII; Figure 17) [91]. Out of these materials, the 2,7-substituted derivatives (CA-XX and CA-XIX) showed not only good solubility due to the highly twisted structure, but also high PCEs of 16.74% and 14.92%, respectively [91], the former being even higher than for the spiro-OMeTAD reference. On the other hand, the 3,6-substituted carbazole-based HTM CA-XXII exhibited a non-promising PCE of 13.3%.

Wu et al. have recently introduced a carbazole-based HTM including the electron-deficient benzothiadiazole (BT) core (CA-XXIII; Figure 17) [105], which was compared to the previously reported CA-X by Xu et al. [92]. The introduction of a BT unit between biphenyl structures in CA-XXIII effectively enhanced the intermolecular interactions, increasing the charge transport, the hole-mobility and the thermal stability of the material. Furthermore, CA-XXIII exhibited better charge collection and transportation properties than the CA-X derivative. When used as HTM in a PSC device, PCE = 16.87% was achieved for CA-XXIII, higher than for spiro-OMeTAD (15.53%).

(i) Other small-molecules HTMs

**Figure 18:**
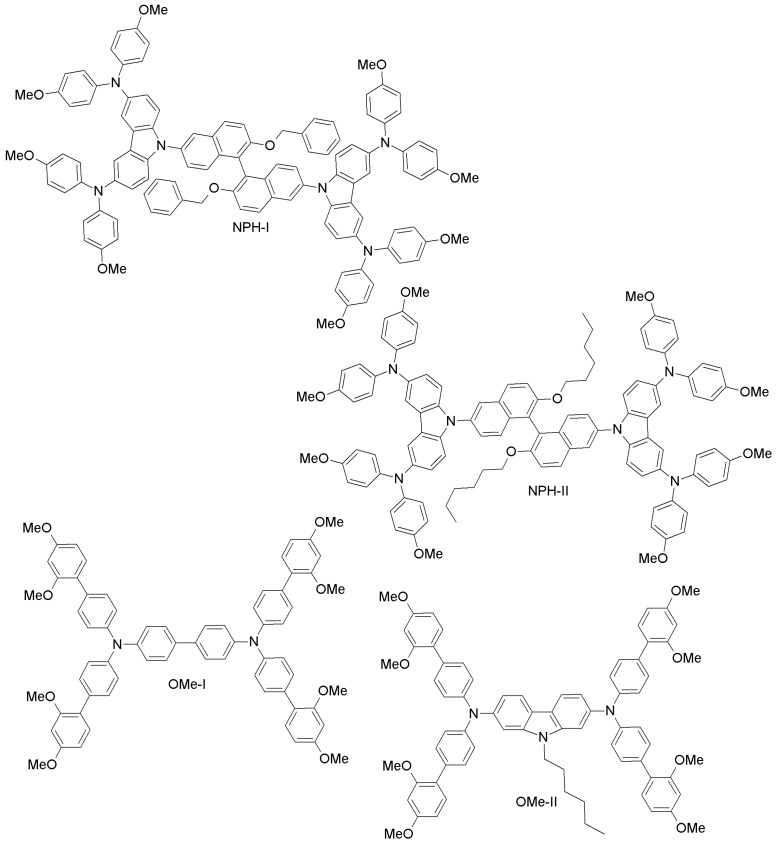
Other organic HTMs.

**Figure 19:**
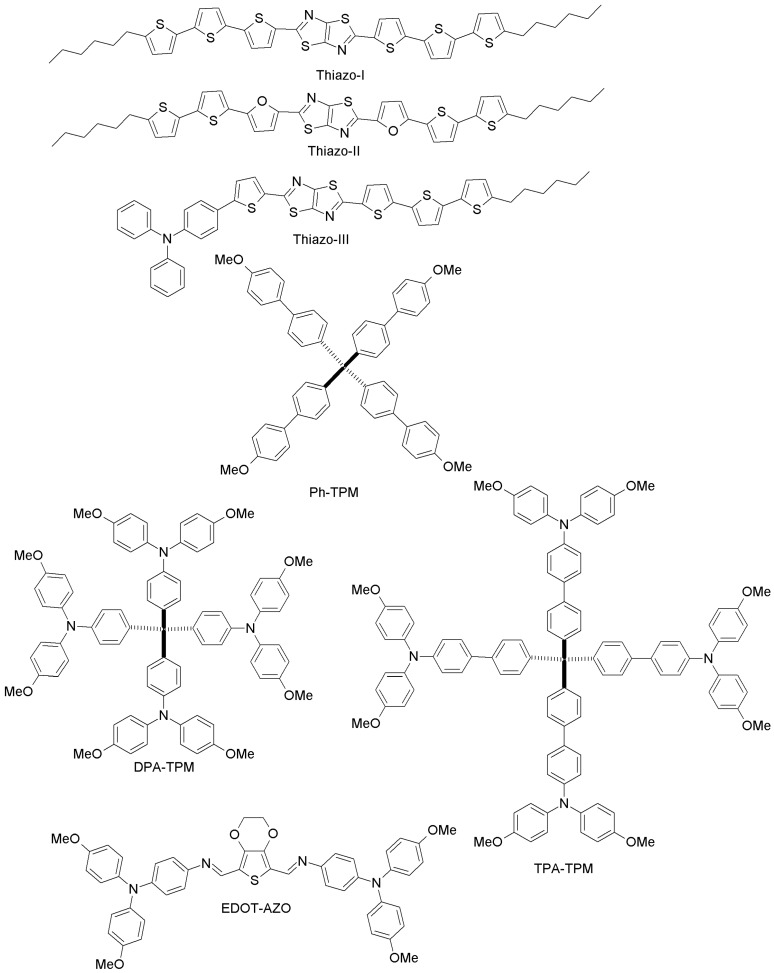
Other organic HTMs (2).

Zong and co-workers have proposed two novel binaphthol-based designs (NPH-I, NPH-II; Figure 18), which differ in the aromatic or aliphatic linkage to binaphthol unit [93]. The electrochemical measurements revealed that the HOMO energy levels of both materials (−5.41 eV and −5.39 eV for NPH-I and NPH-II, respectively) are well-aligned with that of perovskite, and PCE values similar to the spiro-OMeTAD reference were reported. Li et al. have made two different HTMs by changing the π-linker unit (biphenyl vs. carbazole; OMe-I, OMe-II; Figure 18) [94]. PCEs of 18.34% and 16.14% were obtained for OMe-II and OMe-I, respectively. The higher efficiency of OMe-II could be due to its higher hole-mobility (2.26 × 10^−4^ cm^2^V^−1^s^−1^ vs. 7.83 × 10^−5^ cm^2^V^−1^s^−1^ for OMe-I), indicating clearly the promise of carbazole as the core unit of HTMs. Based on the work by Li et al. [94], Nazim and co-workers have recently synthesized three low-cost thiazolo[5,4-d]thiazole-based HTMs (Thiazo-I, Thiazo-II and Thiazo-III; Figure 19) [95].

Their appropriate energy levels, tuned to match those of CH_3_NH_3_PbI_3_, render them suitable to be employed in PSCs, which exhibited a PCE of 10.60%, 4.37% and 8.63% for Thiazo-II, Thiazo-I and Thiazo-III, respectively. In particular, Thiazo-II, with a furan unit in the thiazolo[5,4-d]thiazole-core, provides a good interface with the perovskite film, allowing for ultrafast and complete intermolecular hole transfer from the photoexcited perovskite layer. Three novel tetraphenylmethane(TPM)-arylamine-based hole-transporting materials (anisole, Ph-TPM, and bulky arylamine side groups DPA or TPMA, DPA-TPM and TPA-TPM; Figure 19) were presented by Liu et al. [96]. PSCs based on these three novel HTMs achieved good PCE values of 4.62%, 9.33% and 15.06% respectively, whereas under similar conditions, spiro-OMeTAD exhibited a PCE of 15.49%.

Finally, we want to highlight the work by Petrus et al., who have addressed the issue of reducing HTM synthetic costs by proposing a material with 3,4-ethylenedioxy thiophene (EDOT) as the central core (EDOT-AZO; Figure 19) [97]. EDOT-AZO was synthesized by a simple one-step Schiff base condensation chemistry of amine and aldehyde of EDOT under ambient conditions using inexpensive precursors. Indeed, currently EDOT-AZO is the least expensive HTM ever reported in the context of PSCs (the cost of EDOT-AZO is only 10 $/g). This work demonstrates how, by adopting simple chemical procedures and cost-effective raw materials, it has been possible to synthesize a high-performance material with only water as the byproduct. When EDOT-AZO was employed as HTM in planar CH_3_NH_3_PbI_3_ PSCs, it led to a performance (PCE = 11%) comparable to that of spiro-OMeTAD-based PSCs (11.9%).

#### 3.1.2. Polymer-Based Hole-Transporters

**Figure 20:**
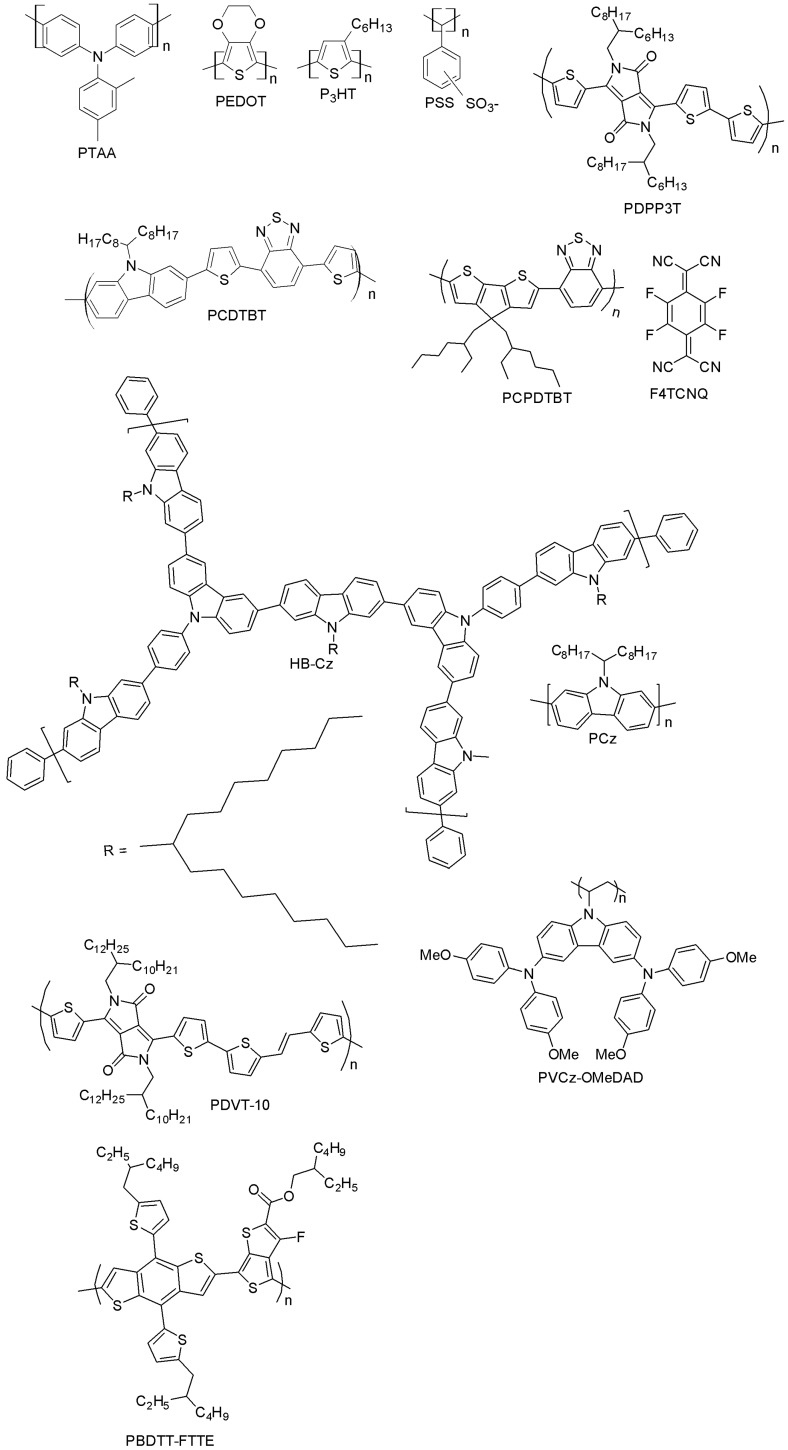
Polymer-based HTMs.

Figure 20 summarizes the polymeric hole-transporting materials that will be surveyed in the rest of this section. An overview of their photovoltaic performance is also presented in Table 2.

Among the polymer-based HTMs, PTAA was the first one tested in PSCs and so far the most efficient, as well. Starting from an earlier work by Seok et al., where PTAA partly infiltrates into a mesoscopic scaffold forming a zig-zag-like structure (the achieved efficiency of the PSC was 12%), an extensive optimization process has been carried out with the utilization of mixed perovskites (MAPbBr_3_/FAPbI_3_), leading to a PCE of over 20% [10,12,47]. The superior performance of PTAA-based PSCs arises from the exceptional hole-mobility of PTAA as compared to other polymers (10^−2^–10^−3^ cm^2^V^−1^s^−1^), as well as from its strong chemical interaction with perovskite. However, the high molecular weight of PTAA does not allow easy infiltration into the pores of the TiO_2_ scaffold. In addition, PTAA is an extremely expensive material (~2000 $/g [106]). Poly(3-hexylthiophene) (P3HT) and PEDOT:PSS are very well-known conducting polymers for organic photovoltaic applications and have been adopted as HTMs in polymer solar cells [107]. When P3HT was used as HTM in a mixed perovskite solar cell (MAPbI_3_/MAPbI_3-x_Cl_x_), an increase in the efficiency from 6.7% (for the traditional MAPbI_3_ perovskite-based cells) [104] up to 13% (for mixed-ion perovskite cells) [108] was obtained. More advanced structures based on P3HT have been recently developed, such as bamboo-structured carbon nanotubes [109] or P3HT-modified carbon nanotube cathodes [110], displaying high efficiency and stability, while keeping the fabrication costs low. PEDOT:PSS has been mostly adopted in inverted planar PSCs with successful outcomes, thanks to the good matching of its work-function with the valence band of the perovskite, good film properties and low-temperature processability. You et al. have reported a 17.1% efficiency in such a PSC [111], while the highest efficiency with PEDOT:PSS (18.1%) so far has been achieved with perovskite and hydrogen iodide (HI) additive [30], which ensured very high quality of the perovskite film. The main well-known drawback of PEDOT:PSS is its hygroscopicity, which limits the chemical stability of PEDOT:PSS-based cells in ambient conditions.

Several other polymers have been tested as HTMs in mesoporous PSCs. Recently, Dubey et al. presented a diketopyrrolopyrrole-based polymer (PDPP3T, poly[{2,5-bis(2-hexyldecyl)-2,3,5,6-tetrahydro-3,6-dioxopyrrolo[3,4-c]pyrrole-1,4-diyl}-alt-{[2,2’:5’,2’’-terthiophene]-5,5’’-diyl}]) that did not decrease the PSC efficiency when used to replace the doped spiro-OMeTAD (12.32% for PDPP3T vs. 12.34% for spiro-OMeTAD) [112]. As an additional attractive feature, the PDPP3T-based device yielded slower device degradation. PCE decreased up to 60.6% of its initial value in 172 h, while for spiro-OMeTAD cells, the loss in PCE was almost 83% of its initial value in the same time.

Stringer et al. have designed a carbazole-based co-polymer, Poly[*N*-9′-heptadecanyl-2,7-carbazole-*alt*-5,5-(4′,7′-di-2-thienyl-2′,1′,3′-benzothiadiazole)] (PCDTBT) [113], which is a well-known donor material in organic BHJ cells. Its applicability as an HTM in PSCs after doping by LiTFSI and TBP was investigated, and a PCE of 15.9%, close to that of spiro-OMeTAD reference, was reported. Its good air stability is due to the low-lying HOMO level (−5.45 eV), lower than in spiro-OMeTAD (in the range of −5.0–−5.22 eV). Thus, PCDTBT-doped HTMs have been used in standard (n-i-p) PSC device architectures, but unfortunately, very low power conversion efficiencies (4.2%) have been obtained [114]. In [115], the authors optimized the thickness and the doping level of PCDTBT in the FTO/c-TiO_2_/mesoporous TiO_2_/(FAPbI_3_)_0.85_ (MAPbBr_3_)_0.15_/PCDTBT/Au structure, reaching an excellent performance (PCE = 15.9%). These results were comparable with those with conventional spiro-OMeTAD-based PSCs (PCE = 7.4%). In one recent report by Yu et al., a co-polymer based on Poly[2,6-(4,4-bis-(2-ethylhexyl)-4*H*-cyclopenta [2,1-*b*;3,4-*b*′]dithiophene)-*alt*-4,7(2,1,3-benzothiadiazole)] (PCPDTBT) has been investigated. Upon doping of 2,3,5,6-tetrafluoro-7,7,8,8-tetracyanoquinodimethane (F4TCNQ) with PCPDTBT, a PCE of 15.1% was reached [115], the highest ever reported for PCPDTBT-based polymers. Zhou and co-workers have recently prepared a hyper-branched carbazole-based polymer (HB-CZ) in one step via the Suzuki coupling reaction and successfully utilized it as a hole-transporting material for a perovskite device [116]. The polymer absorbs in the UV region (which makes it a screen against PSCs’ degradation), has high hole-mobility, a deep HOMO energy level (−5.32 eV) and high LUMO energy (−2.42 eV). HB-CZ-based cells exhibited a PCE of 14.07%, which is higher than reference devices made up of commercially available HTMs such as P3HT (PCE = 9.05%) and polycarbazole (PCz) (6.60%). Liu et al. have in turn designed a highly π-extended copolymer HTM Poly{3,6-dithiophen-2-yl-2,5-di(2-decyltetradecyl)-pyrrolo[3,4-c]pyrrole-1,4-dione-alt-thienylenevinylene-2,5-yl} (PDVT-10), with an impressive hole-mobility of 8.2 cm^2^V^−1^·s^−1^ and good performance in PSCs (PCE = 13.4%) without doping [117], representing one of the highest PCEs reported for dopant-free polymer-based HTMs.

Xu and co-workers have reported a novel carbazole-based non-conjugated polymer (PVCz-OMeDAD), which bears a non-conjugated polyvinyl chain and a hole-transporting OMeDAD unit [118]. The material is obtained by free radical polymerization of vinyl monomer using low-cost raw materials with high reaction yields. The OMeDAD moiety enhances the molecule’s hole-transporting capabilities, and a PCE of 16.09% has been reported, which is significantly better than that of the spiro-OMeTAD-based reference cells (9.62%). Gaml et al. have reported the use of benzodithiophene-based polymer poly[4,8-bis(5-(2-ethylhexyl)thiophen-2-yl)benzo[1,2-b;4,5-b’]dithiophene-2,6-diyl-alt-(4-(2-ethylhexyl)-3-fluorothieno[3,4-b]thiophene-)-2-carboxylate-2-6-diyl)], PBDTT-FTTE, as an HTM in PSCs [119]. After doping PBDTT-FTTE with 3% of diiodooctane, it led to PSCs with similar performance as conventional spiro-OMeTAD-based devices in an inert atmosphere, but with an enhanced fill factor and open-circuit voltage. Diiodooctane-doped PBDTT-FTTE cells exhibited an efficiency of 11.6%, while the PCE of the non-doped PBDTT-FTTE devices was slightly lower (PCE = 10.3%). This highlights very well the importance of diiodooctane doping for PBDTT-FTTE polymers as efficient HTMs.

Further research is mandatory to explore novel polymeric HTMs whose absorption lies beyond that of perovskite, to contribute to the external quantum efficiency of the device. In fact, the so-far proposed HTMs mostly function as charge carriers rather than light absorbers. However, polymeric HTMs offer several drawbacks when thinking of commercialization, such as their polydispersity, which results in more difficult characterization with undefined molecular weight, lower purity, batch-to-batch variation and last, but not least, poor infiltration into the nanostructured material [4,40].

### 3.2. Inorganic Hole-Transporting Materials

The great majority of research on efficient PSCs has been performed using organic hole conductors, despite their challenging syntheses and high purity requirements. For large-scale industrial applications, inorganic p-type HTMs represent a promising, but quite unexplored alternative to the organic ones. They combine high mobility with low fabrication costs and good chemical stability [120]. However, to date, only a few examples of inorganic HTMs are present in the literature, due to the limited choice of suitable materials [5]. Moreover, the solvents used for inorganic HTMs can partially dissolve the perovskite, thus compromising the device stability.

In this section, we will review several inorganic HTMs in PSCs, with a stronger focus on the latest and most significant research results related to mesoscopic PSCs. In fact, in the literature, the reader can find several other good reviews on this topic already [4,5,120,121,122].

Nickel oxide (NiO) is probably the most widely-studied and most promising inorganic HTM at present, leading to the highest values of efficiency and stability. The interest in NiO lies in the fact that it is a very robust, abundant and low-cost material. It has been employed in different device configurations, such as inverted mesoscopic PSCs [123,124,125,126], mesoporous carbon electrode-based PSCs [127,128,129,130] and inverted planar PSCs [131,132,133,134,135,136,137,138,139,140,141,142].

In the papers reported above, several treatments have been proposed to enhance the quality and the homogeneity of NiO thin layers, such as sputtering and pulsed-laser deposition [124,125,135], with an optimized PCE of 17.3%. In other examples, NiO has been modified by doping (e.g., Cu [141]) or combined with small metals (e.g., Li, Mg) or PEDOT. Among the referenced works on inverted planar PSCs, it is worth highlighting the article by Chen et al., where a large-aperture PSC area with 15% PCE is presented [132].

Within the “fully-printed PSCs”, an attractive concept when thinking of industrial applications, NiO has been selected by Wang et al. as HTM with a multiporous-layered structure (PCE = 15.03%) [123,129,130].

**Figure 21:**
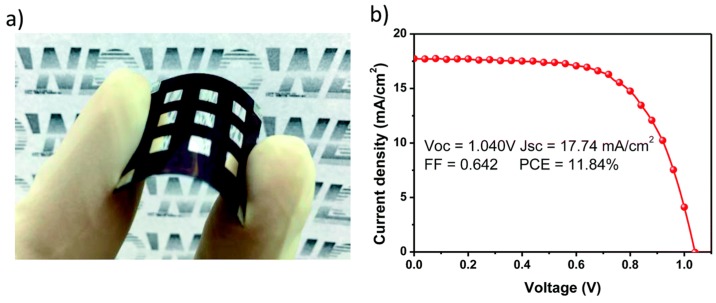
(**a**) Flexible PSC employing NiOx as HTM; (**b**) figures of merit of the flexible PSC (reproduced with permission from [143], published by the Royal Society of Chemistry).

Very recently, a low-temperature solution-processed NiOx thin film was first employed as a HTM in both inverted (p-i-n) planar and regular (n-i-p) mesoscopic organic-inorganic hybrid PSCs [143]. The greatest achievement of this work was to introduce a presynthesized NiOx directly deposited on top of the perovskite film (in n-i-p structures) without decomposing the perovskite itself. After proper treatment of the NiOx thin film, a promising power conversion efficiency of 15.9% with negligible hysteresis (Table 3) was obtained for inverted planar PSCs, and 11.8% was obtained for the flexible devices (Figure 21). This work paves the way to all inorganic PSCs, for efficient, stable and low-cost devices.

Copper (Cu) has been also successfully employed to make low-cost Cu-based inorganic HTMs, which can be deposited via solution processing methods with good pore filling.

The first one, explored by Kamat et al., was based on copper iodide (CuI), with an overall efficiency of over 6% [144]. This modest performance is mainly attributed to the low V_OC_. The most interesting examples of this class of HTM appeared after a couple of years and included cupric oxide (CuO), cuprous oxide (Cu_2_O) and copper thiocyanate (CuSCN). These wide-bandgap semiconductors have high conductivity, suitable energy level and good transparency. These properties may represent a paradigm shift particularly for perovskite-based tandem solar cells.

Cu_2_O has a narrow band-gap, high hole-mobility, low-cost and it is environmentally friendly [145,146]. Being highly sensitive to the underlying mixture of perovskite precursors and solvents, in [145], the authors deposited Cu_2_O by reactive magnetron sputtering. The corresponding PSCs show a PCE of 8.93% and relatively good stability of over 30 days in air.

The first remarkable work on CuSCN HTM in PSCs was reported in 2014 by Grätzel et al. [147]. A PSC with 12.4% efficiency was presented, with a reference cell without HTM displaying only 9% efficiency. The improved performance exhibited with the CuSCN HTM was due to the efficient charge extraction and collection from the excited perovskite (CH_3_NH_3_PbI_3_ in this case) to TiO_2_ and CuSCN, respectively, and then to the corresponding electrodes.

**Figure 22:**
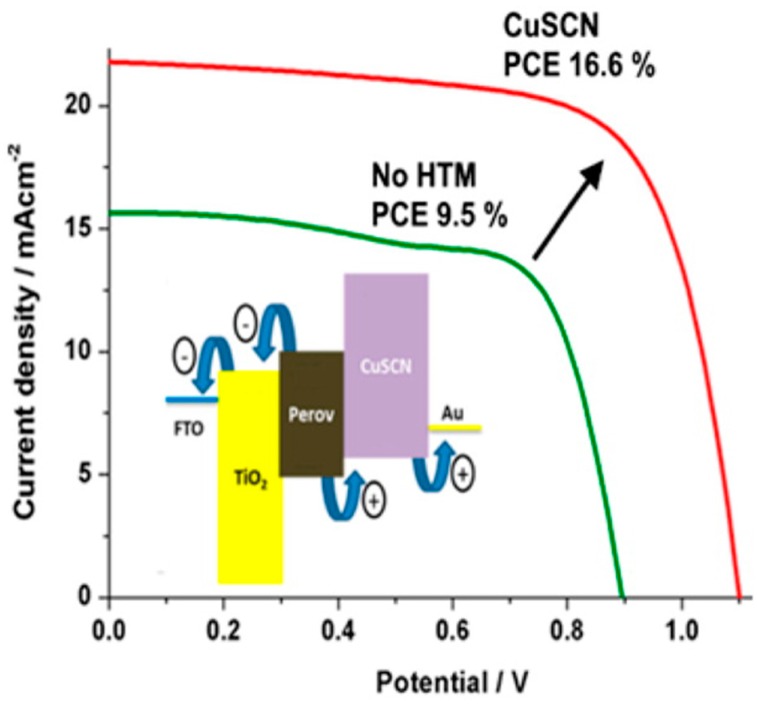
Current-voltage curves for PSC with copper thiocyanate (CuSCN) HTM and without any HTM (reproduced with permission from [148], published by the American Chemical Society).

Nazeeruddin et al. recently reported a PSC based on the mixed (FAPbI_3_)0.85(MAPbBr_3_)0.15 composition, in combination with CuSCN HTM with over 16% PCE and a remarkable monochromatic incident photon-to-electron conversion efficiency of 85% [148]. Under similar conditions, the device without CuSCN exhibited a PCE of 9.5%, mostly attributed to a significant decrease in the short-circuit current (from 21.8 mA/cm^2^ down to 15.64 mA/cm^2^) and open-circuit voltage (from 1100 mV down to 900 mV) (Figure 22). The authors attribute the higher Jsc with CuSCN to the effective charge transfer between perovskite and CuSCN, followed by the fast hole transport through CuSCN to the Au.

A higher PCE has been reported by Jung et al. by using a low-temperature solution-processed CuSCN, with a highly stable crystalline structure [149]. In addition, the authors also demonstrated a high stability of CuSCN-based cells as compared to spiro-OMeTAD-based ones. While the PSC fabricated with spiro-OMeTAD degraded to 25% of the initial PCE after annealing for 2 h at 125 °C in air under 40% average relative humidity, the CuSCN-based PSCs maintained approximately 60% of the initial value. This proves that, by using CuSCN as HTM in PSCs, high efficiency and improved thermal stability can be simultaneously achieved.

The stability issue of spiro-OMeTAD-based PSCs was addressed also by Di Carlo et al. in their work on reduced graphene oxide (RGO) as HTM in mesoscopic PSCs [150]. A simple and reproducible method for the graphene oxide reduction is described, and mesoscopic PSCs with RGO are presented, together with control structures employing spiro-OMeTAD. Excellent endurance properties (1987-h shelf-lifetime) were demonstrated for RGO-cells, confirmed by long-term stability tests including light-soaking experiments. Moreover, employing RGO as HTM resulted in considerably lower fabrication costs compared to spiro-OMeTAD.

Other inorganic HTMs for PSCs include the transition metals oxides MoO_3_ and VO_x_. In spite of the advantages of nontoxicity (MoO_3_), air stability (MoO_3_) and high work-function (VO_x_), their adoption has not resulted in very efficient devices [122]. In fact, these HTMs have a very poor surface morphology, which makes the quality of the deposited perovskite films extremely low. For these reasons and because no relevant progress has been subsequently reported about MoO_3_/VO_x_ HTMs in PSCs, we will not discuss them in further detail. The interested reader may refer to [120] or [122] for a comprehensive overview of transition metal oxides HTMs.

### 3.3. Hybrid Hole-Transporters

The high flexibility of photovoltaic contacts is an essential prerequisite for bringing PSCs closer to the industrial fabrication, e.g., by R2R printing technology. In addition, in order to produce cost-effective solar cells, it is necessary to reduce the number of vacuum steps in the fabrication and to replace the Au top-electrode with a less expensive material. Furthermore, the migration of Au from the back-hole contact through the entire PSC is responsible for relevant performance losses, thus stressing even more the need for alternative metallic contacts.

To overcome such limitations of current contacts in PSCs, a novel concept of hybrid HTM has been reported in the literature, which relies on carbon nanotubes. The function of the nanotubes in different architectures ranges from a hole-transporter additive and an interface modifier, to a hole-transporting system, as well as a charge-selective electrode. The inherent resilience, low-cost and stability of nanotubes makes them an ideal candidate to replace the traditional contacts in PSCs.

**Figure 23:**
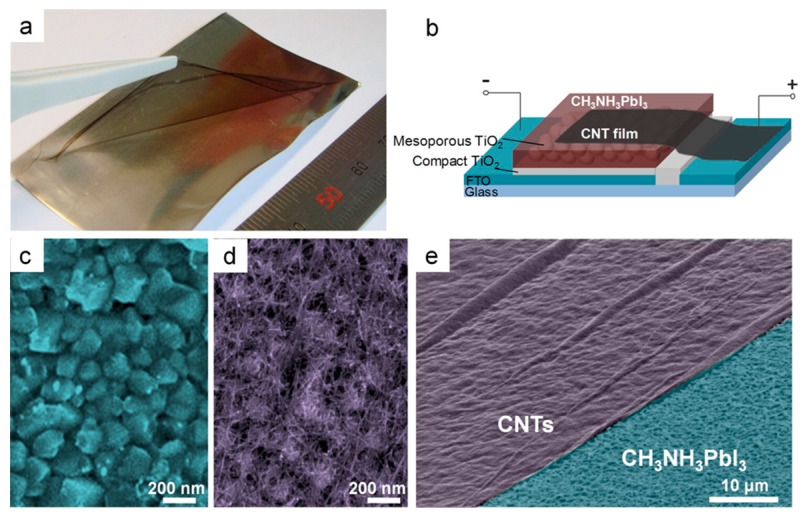
(**a**) Photo of freestanding CNT film lifted by tweezers; (**b**) mesoscopic CH_3_NH_3_PbI_3_ perovskite solar cell with CNT film electrode; (**c**) top view SEM images of CH_3_NH_3_PbI_3_ perovskite substrate before and (**d**) after CNT transfer; (**e**) tilted SEM image of CH_3_NH_3_PbI_3_ perovskite substrate (blue) partly covered by CNT film (purple) (reproduced with permission from [151], published by the American Chemical Society).

Initially, free-standing films of single-walled and double-walled carbon nanotubes (CNTs) were tested in mesostructured PSCs by Li and co-workers [151], with a dual-function of hole transporter and cathode at the same time. The devices performed poorly (PCE = 6.3%), due to the relatively high resistance of the nanotube films and to the lack of charge selectivity (Figure 23). The performance significantly increased upon introducing a spiro-OMeTAD layer, which allowed achieving a PCE of 9.9%. This work demonstrated that it is possible to eliminate the metal top electrode, such as Au or Ag.

Carbon nanotubes have been also used as spiro-OMeTAD additives in PSCs [152,153], as well as mixed with spiro-OMeTAD, originating a composite HTM [153,154], or wrapped in polymers [82,153].

**Figure 24:**
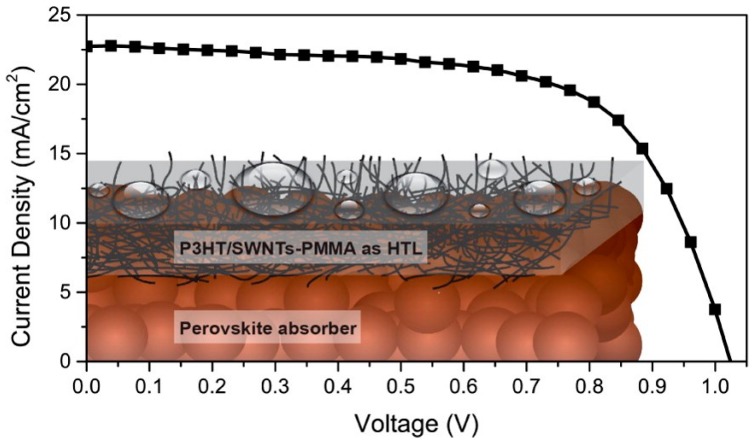
Schematic illustration of the perovskite solar cell with carbon nanotube/polymer composite as the non-hygroscopic hole-transporting structure, referred as HTL in the figure (reproduced with permission from [155], published by the American Chemical Society).

Snaith et al. achieved significant thermal/moisture stability improvements in PSCs by replacing the traditional organic HTM with polymer-functionalized (P3HT, PTAA) single-walled CNT embedded in an insulating polymer matrix (polymethylmethacrylate (PMMA)) [155]. The key intuition was that the HTM has a protective effect on the perovskite structure, by shielding it from atmospheric moisture, responsible for quick degradation. This also explains why a non-hygroscopic HTM like the system proposed in [155] (Figure 24) allowed good stability of the photovoltaic devices while being suitable for printing technology. This work demonstrates that, when designing new HTMs for PSCS, it is crucial to take into account not only the electronic characteristics of the HTM, but also its hydrophobicity/permeability properties. In fact, a non-hygroscopic HTM is highly recommended because of its resilience against thermal stressing and moisture ingress.

**Figure 25:**
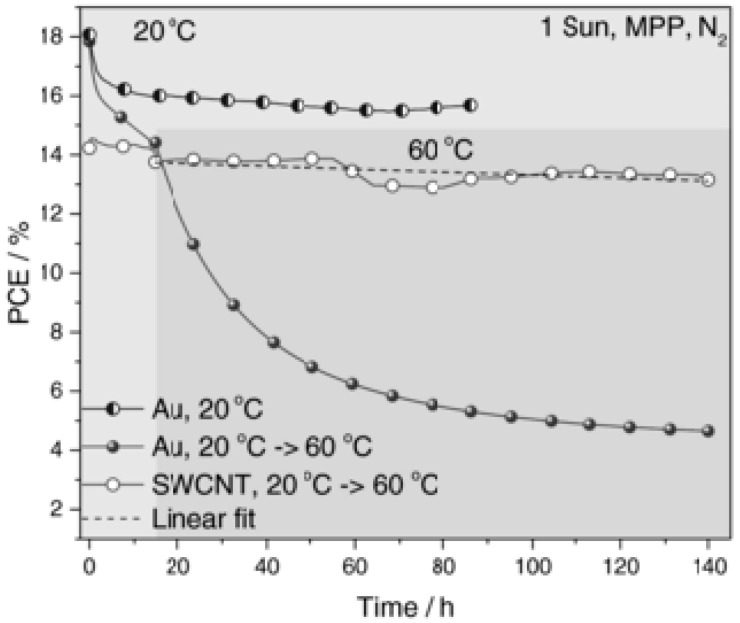
Maximum power point (MPP) tracking of Au and SWCNT-contacted devices at high temperatures (reproduced with permission from [156], published by John Wiley & Sons, Inc.).

Aitola et al. presented an interesting system composed of a single-walled carbon nanotube thin film with a drop-cast spiro-OMeTAD. Such an SWCNT:spiro-OMeTAD hybrid composite works as an HTM-counter electrode (CE) system, i.e., the solar cell does not need an evaporated metal electrode. The record efficiency achieved by Aitola et al. by the PSC with HTM-CE was 15.5%, while the control cell with traditional spiro-OMeTAD and the evaporated Au electrode had 18.8% record efficiency. This study proves that a potentially low-cost HTM-CE composite relying on single-walled CNTs can be successfully deposited directly onto the perovskite layer (FAPbI_3_:MAPbBr_3_ in this case) of a PSC and yield very acceptable performance. More recently, high long-term stability at elevated temperatures of PSCs was achieved with HTM-CE manufactured by a simple press transfer of the CNT film on the mixed ion perovskite absorber and by infiltrating the CNT film with spiro-OMeTAD [156]. The experiments conducted at 60 °C in N_2_ atmosphere and one Sun showed only a modest linear efficiency loss for the devices (Figure 25). Their lifetime, defined as the point where 20% of the initial PCE is lost, has been estimated as 580 h. At the same time, the authors fabricated standard PCSs with a Au back contact, which exhibited a dramatic and rapid efficiency loss due to the Au ion migration in the structure [156].

## 4. Concluding Remarks and Future Perspectives

In spite of the exciting and rapid development of perovskite solar cells, intense research efforts are still needed in order to overcome the main bottlenecks to their commercialization, namely stability, upscaling and cost-effectiveness. Printing and coating techniques similar to mass production methods (e.g., roll-to-roll fabrication) would allow fabricating large-scale devices, thus providing a huge step towards the successful manufacturing and commercialization of PSC-based technologies.

Solution-processable HTMs play a big role in this respect and should be judiciously designed to allow achieving high device efficiencies and at the same time enhanced stability and cost effectiveness compared to the obvious choice, spiro-OMeTAD. In fact, while spiro-OMeTAD is still currently needed to achieve the highest efficiency, it has mediocre hole-mobility (and thus, it requires hygroscopic dopants) and a high cost.

Ideally, HTMs should exhibit the following key properties:

- Appropriate ionization potentials are essential to guarantee efficient transfer of the photoexcited holes from the perovskite layer to the HTM.
- Low electron affinities are required to avoid electron recombination at the perovskite|HTM interface.
- HTMs should also be highly transparent, low-cost and present good film-forming properties at the same time, which ensure smooth and pinhole-free coverage.
- High mobility: Doping of the organic HTMs is in most cases necessary to ensure high cells performance, but it is usually responsible for significant degradation processes compared to pristine HTMs. To enhance the stability, it would be ideal to avoid any dopant to the pristine HTM. This requires the HTM to have high mobility. Research efforts in this direction must continue, starting from the promising achievements about the highly order columnar design concept as a strategy to facilitate the mobility of charge-carriers by face-on arrangement. This was the case of material Triazatrux-VII, which demonstrated how to enhance the hole-mobility to achieve exceptional device performance.
- If the use of dopants cannot be avoided because of the poor hole-mobility of the pristine HTM, hydrophobic dopants or air-stable additives should be chosen, as alternatives to the highly unstable dopants commonly used at present (LiTFSI, TBP, FK 209).

In the last few years, a big number of novel HTMs has been proposed, mainly based on organic small-molecules. Fewer examples include polymers, organometallic, inorganic and hybrid HTMs. In this work, we have reviewed the most recent advances in HTM design and synthesis. In particular, we have looked at this topic from a synthetic chemist’s prospective, highlighting the correlation between the HTM molecular design and the performance of the corresponding PSCs. We believe that in this way, we will provide inspiration to many scientists in the field, to design the next generation of HTMs for low-cost, efficient and air-stable perovskite photovoltaics.

In the context of small-molecule HTMs, different synthetic strategies have been presented in the literature, with the idea of simplifying the intricate synthetic steps required for spiro-OMeTAD, which directly affect its prohibitive cost (500 $/g). Many of the recent small-molecule-based HTM designs, mostly containing di- and tri-arylamine groups, successfully performed in PSCs, similarly or even better than spiro-OMeTAD (see Table 1).

Trux-II and Triazatrux-VII are promising organic HTMs with enhanced efficiency and stability with respect to spiro-OMeTAD. They are based on truxene and triazatruxene core, respectively, and can ensure highly-efficient cells when used in pristine form. XPP, BTHIO, spiro-FL-III and SPI-FL-MP-2PA are also interesting examples of HTMs with high efficiency and stability when adopted in PSCs. Further investigation and optimization of these designs may be very beneficial for the future commercialization of perovskite technology.

At present, the least expensive HTM remains EDOT-OMeTPA (10 $/g), which displays nice performance in photovoltaics, on par with that of spiro-OMeTAD. FDT (60 $/g) is a more recent example of a low-cost HTM with excellent performance in PSCs. Many possibilities to further engineer and tune the FDT-core should still be explored.

As for polymers, the most successful HTM remains PTAA, adopted in the structure displaying the current world efficiency record (22.1%). However, PTAA is extremely expensive (2000 $/g). Another high-performance polymeric HTM is PVCz-OMEDAD, which led to devices with a PCE of around 16%. A common drawback to all polymeric HTMs is the limited reproducibility in terms of molecular weight and batch-to-batch variation. This is why most of the research work is dedicated to novel small-molecule HTM designing, due to their low-cost, high reproducibility, easier synthesis and tunable optical and electrochemical properties.

Recently, inorganic HTMs have emerged as a valid class of materials to guarantee high device stability. Particularly, NiO seems to be a suitable candidate for high-performance PSCs. Nevertheless, the examples of inorganic HTMs presented in the literature are still limited. Bigger research efforts are required to identify novel materials with suitable chemical and electronic features to ensure high performance PSCs.

In the last year, a new concept of hybrid HTMs, based on carbon nanotubes (CNTs), has appeared. Because of their inherent chemical and mechanical stability, CNTs are a promising and more stable alternative to conventional materials and can be successfully used in their pristine form. Nevertheless, the efficiencies of CNT-based perovskite solar cells do not yet compete with other types of HTMs. Further improvements in the power conversion efficiencies could make them a highly competitive and convenient option for highly performing and stable PSCs.

**Figure 26:**
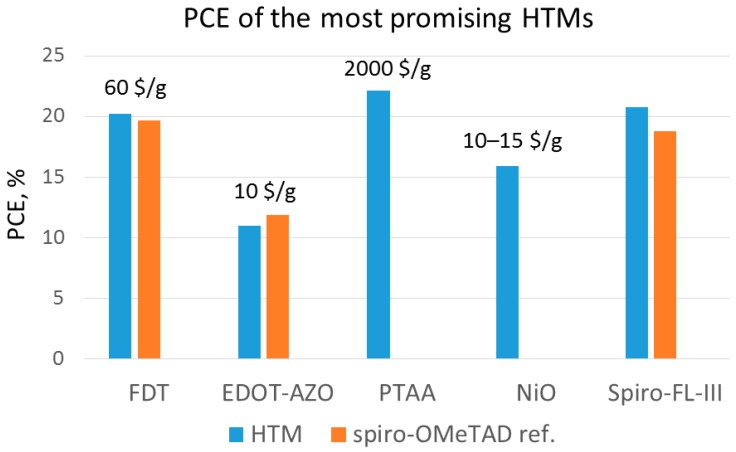
Summary of the most efficient HTMs discussed in this work, together with their cost (when available).

In conclusion, it is not possible to identify a unique direction for the design of the perfect HTM. We have highlighted the most promising molecules so far (see Figure 26) and their structure-property relationship in PSCs. However, since most of the reports refer to MAPbI_3_ perovskite and recently the highest efficiencies have been achieved with mixed ion perovskites, the effectiveness of the different HTMs should be assessed in similar conditions for a fair comparison. The lack of a systematic report and the limited available data do not allow this.

Further and deeper understanding is paramount for further rational design, in addition to the typically used trial-and-error approach. In particular, we want to emphasize the role of photophysics and opto-electronics, which can help with understanding how to optimize the charge extraction and recombination processes by fine-tuning of the molecular structures. Computational methods can also offer a valid support in modelling the phenomena taking place in HTMs and at their interface with perovskite. The tight multidisciplinary collaboration between chemists, physicists and device engineers is the key for future breakthroughs in HTM research for low-cost, scalable and highly performing PSCs.

## Figures and Tables

**Table 1:** Summary of the photovoltaic performance (PCE) for each of the small-molecule HTMs reviewed in this work. For comparison, the PCE of the corresponding reference cells containing spiro-OMeTAD (fabricated and characterized in identical conditions) is also reported. TPA, triphenylamine.

Category	HTM	PCE (%), HTM	PCE (%), Spiro-OMeTAD	Reference
Pyrene-based	PY-1	3.3	12.70	[55]
	PY-2	12.3	12.70	[55]
	PY-3	12.4	12.70	[55]
Truxene-core	Trux-I	18.6 (10.2 [56])	16.0 (9.5 [56])	[57]
	Trux-II	13.4	9.50	[56]
	Triazatrux-I	8.9	17.10	[58]
	Triazatrux-II	17.7	17.10	[58]
	Triazatrux-III	15.8	17.10	[58]
	Triazatrux-IV	11.5	17.10	[58]
	Triazatrux-V	8.88	19.01	[59]
	Triazatrux-VI	14.87	19.01	[59]
	Triazatrux-VII	19.03	19.01	[59]
Phenothiazine-based	PH-I	2.10	17.70	[60]
	PH-II	17.60	17.70	[60]
Acridine-, thiophene-, biphenyl-, bithiophene-, tetrathiophene-, difluorobenzene, and phenyl-based	AC-I	16.42	16.26	[61]
	Thio-I	9.05	8.83 (15.63) ^1^	[62]
	Thio-II	15.13	8.83 (15.63) ^1^	[62]
	BPH-I	13.27	16.81	[63]
	BPH-II	16.42	16.81	[63]
	BTHIO	19.40	18.80	[64]
	TETRATH-I	18.13	17.80	[65]
	TETRATH-II	17.3	17.80	[65]
	TETRATH-III	15.7	17.80	[65]
	TETRATH-IV	9.7	17.80	[65]
	DFTAB	10.4	15	[66]
Triazine-based	TRIAZ-I	12.5	13.45	[67]
	TRIAZ-II	10.9	13.45	[67]
	TRIAZ-III	13.2	13.8	[68]
	TRIAZ-IV	12.6	13.8	[68]
Benzotrithiophene- and squaraine-based	BZTR-I	16	18.1	[69]
	BZTR-II	17	18.1	[69]
	BZTR-III	18.2	18.1	[69]
	BZTR-IV	19	18.9	[70]
	BZTR-VHYX	18.2	18.9	[70]
	SQ-H	14.74	15.33	[71]
	SQ-OC_6_H_13_	14.73	15.33	[71]
Fluorene- and spiro-fluorene-based	FL-I	17.8	18.4	[72]
	FL-II	16.73	14.84	[73]
	FL-III	17.25	16.67	[74]
	FL-IV	15.90	16.67	[74]
	FL-V	14.52	17.88	[75]
	FL-VI	15.09	17.88	[75]
	FL-VII	9.15	17.88	[75]
	FL-VIII	16.79	17.88	[75]
	FL-IX	16.45	17.88	[75]
	Spiro-FL-I	19.8	20.8	[76]
	Spiro-FL-II	13.6	18.8	[77]
	Spiro-FL-III	20.8	18.8	[77]
	XDB	5.4	5.5	[78]
	XOP	15.0	5.5	[78]
	XMP	16.5	5.5	[78]
	XPP	17.2	5.5	[78]
	FDT	20.2	19.7	[39]
	SPI-BI	17.77	18.25	[79]
	SPI-TH	19.96	18.25	[79]
	SPI-TRI	19.47	18.25	[79]
	SPI-FL-MM-3PA	13.46	14.98	[80]
	SPI-FL-MP-3PA	15.59	14.98	[80]
	SPI-FL-MM-2PA	12.56	14.98	[80]
	SPI-FL-MP-2PA	13.43	14.98	[80]
Carbazole-based	CA-I	12.3	12.17	[81]
	CA-II	8.5	10.2	[82]
	CA-III	10.2	10.2	[82]
	CA-IV	13.28	15.23	[83]
	CA-V	14.79	15.23	[83]
	CA-VI	13.86	15.23	[83]
	CA-VII	4.53	5.10	[84]
	CA-VIII	0.19	5.10	[84]
	CA-IX	7.6	10.2	[85]
	CA-X	9.8	10.2	[85]
	CA-XI	10.96	13.76	[86]
	CA-XII	12.61	13.76	[86]
	CA-XIII	13.0	13.76	[86]
	CA-XIV	11.4	12.0	[87]
	CA-XV	13.1	12.0	[87]
	CA-XVI	17.8	18.6	[88]
	CA-XVII	17.81	18.59	[89]
	CA-XVIII	12.42	14.32	[90]
	CA-XIX	14.92	15.01	[91]
	CA-XX	16.74	15.01	[91]
	CA-XXI	0	15.01	[91]
	CA-XXII	13.30	15.01	[91]
	CA-XXIII	16.87	15.53	[92]
Other small molecules (Naphthalene (NPH), di- and tetra-phenylmethane (DPA-TPM, TPA-TPM), and ethylene dioxythiophene (EDOT))	NPH-I	10.05	10.06	[93]
	NPH-II	8.66	10.06	[93]
	OMe-I	18.34	-	[94]
	OMe-II	16.14	-	[94]
	Thiazo-I	10.60	-	[95]
	Thiazo-II	4.37	-	[95]
	Thiazo-III	8.63	-	[95]
	Ph-TPM	4.62	15.49	[96]
	DPA-TPM	9.33	15.49	[96]
	TPA-TPM	15.06	15.49	[96]
	EDOT-AZO	11.0	11.9	[97]

^1^ When the spiro-OMeTAD concentration is increased up to 73 mg mL^−1^.

**Table 2:** Overview of the photovoltaic performance (PCE) of the polymeric HTMs presented in this work. For comparison, the performance of reference cells fabricated under identical conditions is also shown. When not otherwise specified, the reference HTM is spiro-OMeTAD. For some polymers (hyper-branched carbazole-based polymer (HB-CZ) and PDVT-10), a different HTM is used for comparison, as indicated in paren thesis.

HTM	PCE (%), HTM	PCE (%), Reference	Reference
PTAA	22.1	-	[12]
P3HT	13.0		[108]
PEDOT:PSS	18.1		[30]
PDPP3T	12.32	12.34	[112]
PCDTBT	15.9	16.6	[113]
PCPDTBT	15.1	-	[115]
HB-CZ	14.07	9.05 (P3HT), 6.60 (PCz)	[116]
PDVT-10	13.4	11.28 (PTAA)	[117]
PVCz-OMeDAD	16.09	9.62	[118]
PBDTT-FTTE	11.6	10.3	[119]

**Table 3:** Device performance of the NiO*_x_*-based inverted planar PSC measured under simulated Air Mass (AM) 1.5 conditions [143].

Scan Direction	V_OC_ (V)	J_SC_ (mA/cm^2^)	FF (%)	PCE (%)
Forward	1.03	20.58	74.7	15.89
Reverse	1.03	20.66	74.2	15.90
